# The Effects of Aging on the Molecular and Cellular Composition of the Prostate Microenvironment

**DOI:** 10.1371/journal.pone.0012501

**Published:** 2010-09-01

**Authors:** Daniella Bianchi-Frias, Funda Vakar-Lopez, Ilsa M. Coleman, Stephen R. Plymate, May J. Reed, Peter S. Nelson

**Affiliations:** 1 Divisions of Human Biology and Clinical Research, Fred Hutchinson Cancer Research Center, Seattle, Washington, United States of America; 2 Department of Pathology, University of Washington, Seattle, Washington, United States of America; 3 Department of Medicine, University of Washington, Seattle, Washington, United States of America; The University of Akron, United States of America

## Abstract

**Background:**

Advancing age is associated with substantial increases in the incidence rates of common diseases affecting the prostate gland including benign prostatic hyperplasia (BPH) and prostate carcinoma. The prostate is comprised of a functional secretory epithelium, a basal epithelium, and a supporting stroma comprised of structural elements, and a spectrum of cell types that includes smooth muscle cells, fibroblasts, and inflammatory cells. As reciprocal interactions between epithelium and stromal constituents are essential for normal organogenesis and serve to maintain normal functions, discordance within the stroma could permit or promote disease processes. In this study we sought to identify aging-associated alterations in the mouse prostate microenvironment that could influence pathology.

**Methodology/Principal Findings:**

We quantitated transcript levels in microdissected glandular-adjacent stroma from young (age 4 months) and old (age 20–24 months) C57BL/6 mice, and identified a significant change in the expression of 1259 genes (p<0.05). These included increases in transcripts encoding proteins associated with inflammation (e.g., *Ccl8*, *Ccl12*), genotoxic/oxidative stress (e.g., *Apod*, *Serpinb5*) and other paracrine-acting effects (e.g., *Cyr61*). The expression of several collagen genes (e.g., *Col1a1* and *Col3a1*) exhibited age-associated declines. By histology, immunofluorescence, and electron microscopy we determined that the collagen matrix is abundant and disorganized, smooth muscle cell orientation is disordered, and inflammatory infiltrates are significantly increased, and are comprised of macrophages, T cells and, to a lesser extent, B cells.

**Conclusion/Significance:**

These findings demonstrate that during normal aging the prostate stroma exhibits phenotypic and molecular characteristics plausibly contributing to the striking age associated pathologies affecting the prostate.

## Introduction

Mammalian aging is associated with molecular, cellular and physiological changes characterized by a deteriorating homeostatic balance associated with the increasing prevalence of neoplasia and other diseases. At the molecular level, aging has been associated with an increase in DNA point mutations, telomere attrition, and alterations in patterns of methylation [1], [2], [3], each of which can disrupt the normal expression and/or function of proteins involved in cellular growth, maintenance of genomic integrity, responses to cellular stress, and inflammation [4], [5]. Replicative senescence (RS)—associated with telomere erosion, and stress-induced premature-senescence (SIPS)—induced by oxidative stress, oncogene activation or DNA damage, have also been linked to the aging process (Reviewed in [6], [7]). Paradoxically, while strong evidence supports cellular senescence as a protective mechanism that inhibits unrestrained cell proliferation and carcinogenesis in the young, it may favor the development of neoplasia and other pathology in the elderly [8]: several studies have demonstrated that concomitant with the loss of ability to replicate, senescent cells secrete an assortment of growth factors, inflammatory cytokines and extracellular matrix proteins that together create a local tissue microenvironment capable of inducing inflammation and promoting the growth of initiated or preneoplastic cells [9], [10], [11].

In man, many of the most common diseases involving the genitourinary tract are strongly associated with advanced age. These include benign hyperplasia of the prostate gland (BPH)—30% of men over age 50, the spectrum of Lower Urinary Tract Symptoms (LUTS), and prostate adenocarcinoma. Autopsy studies indicate that prostate cancer incidence approaches 60–70% in the 8th and 9th decade of life [12], [13]. A relationship between aging and inflammation, hyperplasia and neoplasia of prostatic tissue has also been observed in rodents and dogs [14], [15], [16], [17]. Overall, the etiology of these diseases remains poorly defined despite causing substantial morbidity and mortality in the population.

The prostate gland is composed of secretory luminal epithelium, basal epithelium, neuroendocrine cells and cell types comprising a supporting stroma. The stroma in the human and rodent prostate gland consists primarily of smooth muscle cells and fibroblasts with rarer populations of endothelium, nerve cells, and infiltrating inflammatory cells [18], [19]. The tissue microenvironment plays important roles in producing a spectrum of autocrine/paracrine factors as well as structural molecules that maintain normal cell behavior and organ homeostasis [20], [21], [22]. Accumulating evidence points towards a role for aging-related changes in constituents comprising the prostatic stroma in promoting prostate epithelial cell growth, though most of this evidence is derived from *ex vivo* experimental systems. *In vitro* studies of fibroblasts isolated from prostates of older men were less able to suppress epithelial cell proliferation than fibroblasts isolated from the prostates of younger men [23]. When co-cultured with premalignant prostatic epithelial cells, senescent prostatic fibroblasts promote epithelial cell growth, and this proliferative response is in part due to the overexpression of secreted paracrine-acting factors [11]. These findings suggest that alterations in the prostate microenvironment, mediated by events associated with stromal aging and/or senescence, permit and/or promote epithelial responses that contribute to organ pathologies.

The objective of this study was to systematically define and quantitate histological and molecular features of the prostatic microenvironment that associate with normal aging *in vivo*. We hypothesized that molecular alterations determined through studies of *in vitro* senescence would be evident in the context of advanced organismal age, supporting a role for this cellular program in prostatic diseases. An inbred mouse strain was selected in order to control for genetic and environmental variables that could confound the interpretation of aging phenotypes. We used expression microarrays to quantitate transcript abundance levels in the stromal compartment of the prostate and evaluated immune cell subtypes and structural features by immunohistochemistry and electron microscopy, respectively.

## Results

### Effects of age on prostate cellular composition and morphology

**Figure 1:**
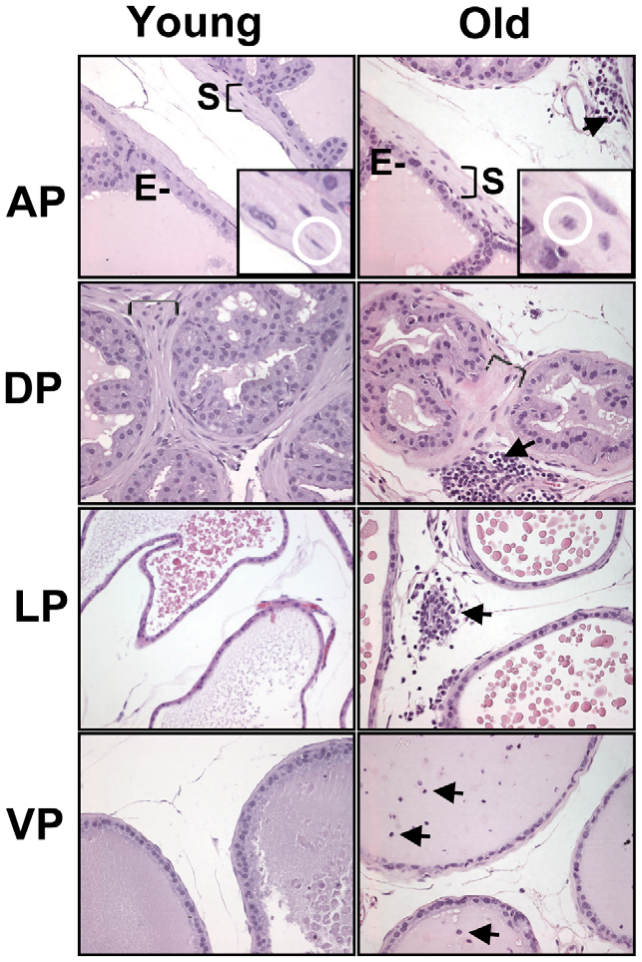
Histological features of prostate glands from young and old mice. Hematoxylin and eosin-stained sections of formalin-fixed prostate tissues from young (4 month-old) and old (24 month-old) mice. E: Luminal epithelium; S: Stroma adjacent to the epithelial cells (glandular-adjacent stroma). Note the thick glandular-adjacent cellular stroma (S, bracket) observed in dorsal and anterior lobe from young and old mice. AP insert: smooth-muscle cells (circled in white) appear less elongated and more rounded in the aged prostate with little evidence of cell orientation. Areas of inflammatory cell infiltration were observed frequently in the prostates of old animals (arrows). AP: anterior prostate; DP: dorsal prostate; LP: lateral prostate and VP: ventral prostate. (Magnification: 20×)

To evaluate the cellular composition of the prostate gland in the context of normal aging, we resected the prostate glands from mice of the C57BL/6 strain aged 4-months, designated young, and 24-months, designated old. We used 4 month-old mice as our young cohort because at this age the males are sexually mature, and therefore less prone to exhibit subsequent changes associated with organogenesis and developmental processes. After dissection the prostates were fixed, embedded in paraffin, sectioned, and stained with hematoxylin and eosin (H&E) for histological studies. Each prostate lobe was individually compared across age groups. Overall, each lobe showed subtle differences in morphology with aging (representative images are shown in **Figure 1**). In contrast to young mice, focal atrophy of a small number of acini as well as epithelial atypia coexisted with morphologically normal acini in old mice. The cellular stroma layer adjacent to the epithelial cells (glandular-adjacent stroma) was generally more disorganized in old animals than in young animals with little evidence of consistent smooth muscle cell directional orientation and evidence of rounding of smooth-muscle and fibroblast cells within the extracellular matrix (**Figure 1**., brackets and inset). Foci of inflammatory infiltrates comprised of cells with characteristic small cell size and little cytoplasm were observed in the interductal stroma and appeared more abundant in the prostates from old animals (**Figure 1**, arrows).

To determine the cell composition of the glandular-adjacent stroma we stained prostate sections from young and old mice by double immunofluorescent staining for smooth-muscle-actin and vimentin (a mesenchymal cell type marker). We determined that 95% of the adjacent stromal cells stained positive for smooth-muscle actin and only 5% stained positive for vimentin (see **Supporting information Figure S1**). Thus, the majority of the glandular-adjacent cellular stroma in the mouse prostate is represented by smooth muscle cells, consistent with prior studies of murine and human prostates [18], [19], [24]. No significant difference in the ratio of cell types expressing these markers was found between young and old prostates. Of note, there was no overlap between smooth muscle actin-positive and vimentin-positive cells, consistent with the lack of a myofibroblast cell type in normal prostate tissue, in both young and old animals.

### Effects of age on gene expression in prostatic stroma

**Figure 2:**
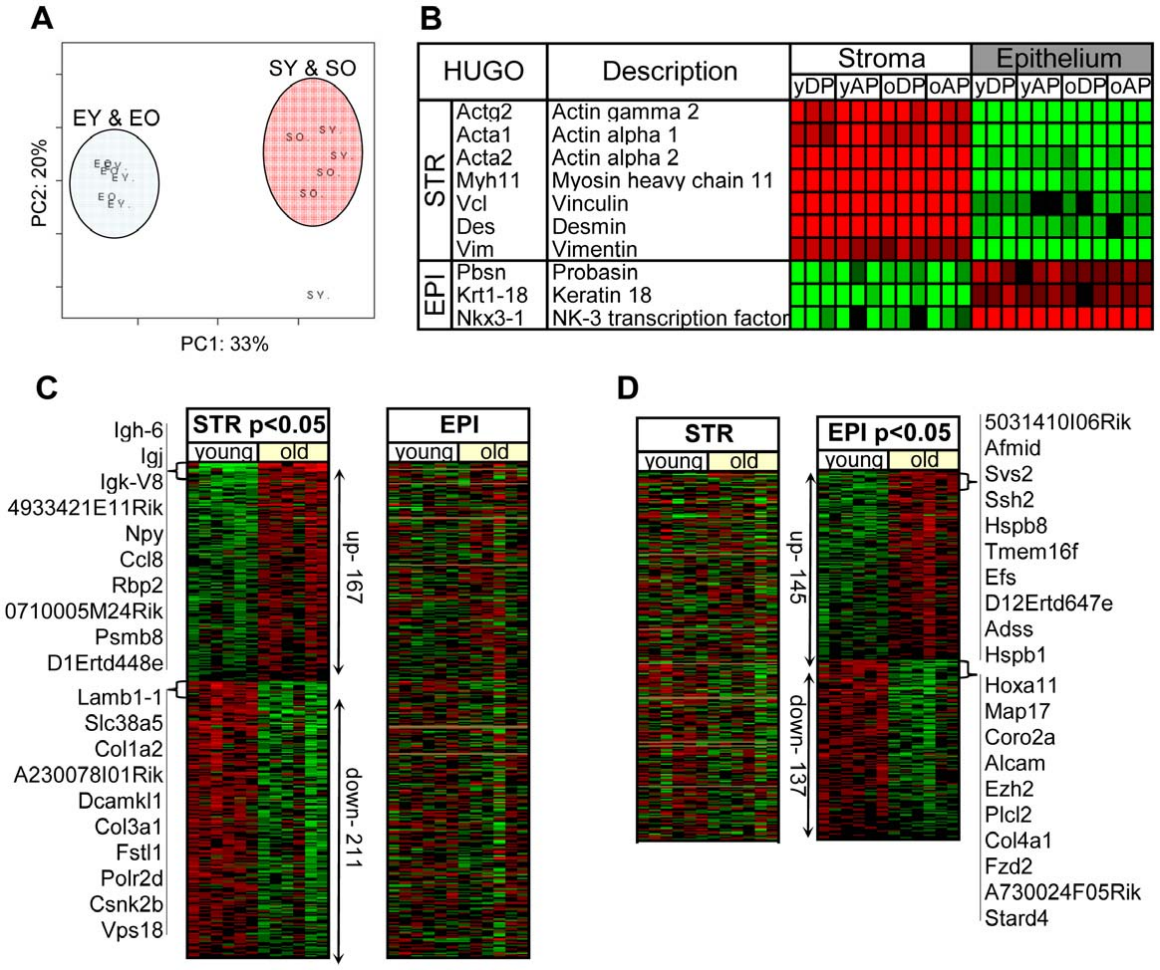
Age and cell type-specific transcript profiles in the mouse prostate. **A**) Principal Component Analysis (PCA) for microdissected dorsal prostate stroma and epithelium from young and old animals. PCA discriminates epithelial and stromal samples. **EO**: old epithelium; **EY**: young epithelium; **SO**: old stroma; **SY**: young stroma. **B**) Transcript abundance levels (Log2 Ratios) obtained from microarray-based measurements for genes known to exhibit preferentially expression in stromal or epithelial cells. Red indicates increased expression; green indicates decreased expression. **C**) Heat map of age-associated transcripts in the prostate stroma (p<0.05) compared to epithelium. Insert: Gene symbols for the 10 most up- and down- regulated genes in the aged stroma. **D**) Heat map of age-associated transcripts in the prostate epithelium (p<0.05) compared to stroma. Insert: Gene symbols for the 10 most up- and down- regulated genes in the aged epithelium. Note the low correlation between the age-related profile of the stroma compared to the epithelium. Heat map colors reflect fold ratio values between sample and reference pool and mean-centered across samples. Columns represent biological replicates from microdissected dorsal and anterior epithelium and stroma for each age group. Rows represent individual genes. Values shown in red are relatively higher than the overall mean; values shown in green are relatively lower than the overall mean; rows shown in brown are genes with no expression values. **STR**: microdissected glandular-adjacent stroma; **EPI**: microdissected luminal epithelium.

A key objective of this study centered on the analysis of aging-related molecular changes in cell types comprising the stromal compartment of the prostate. To evaluate the ability of laser-capture microdissection (LCM) to acquire pure cell populations, we performed a pilot study using LCM to separately isolate luminal epithelial cells and glandular-adjacent stroma from young (n = 5) and old mice (n = 5). We opted to capture cells from the anterior and dorsal lobes since, based on histology, these two lobes have the most abundant cellular smooth-muscle/fibroblastic stroma (**Figure 1**, brackets). In addition, the anterior and dorsolateral lobes have also been reported to be the regions in which prostate intraepithelial neoplasia (PIN) and prostate carcinogenesis begins in murine models [25], [26], [27], [28], [29] and gene expression data indicates that the dorsolateral lobe is most homologous to the peripheral zone of the human prostate, where cancer is most prevalent [30]. We verified cell-type specific purity by analyzing the expression levels of known stromal cell and epithelial cell markers using a customized mouse prostate cDNA array (MPEDB array) [31]. Three biological replicate pools per lobe, representing five 4 month-old and five 24 month-old animals, were generated to facilitate statistical analyses and control for individual variability. As expected, stromal and epithelial transcripts were differentially expressed in the stroma and epithelial samples respectively (**Figure 2A,B**). To further characterize the relationships between the epithelial and stroma samples and between age groups, we performed Principal Component Analysis (PCA) for all the genes in the arrays (**Figure 2A**). PCA clearly grouped a subset of genes that discriminated the epithelial and stroma samples, suggesting that the major differences between samples resulted from the differential expression of large numbers of genes between the stroma and epithelial compartments. These results demonstrate that highly enriched populations of stroma cells can be isolated by microdissection.

**Figure 3:**
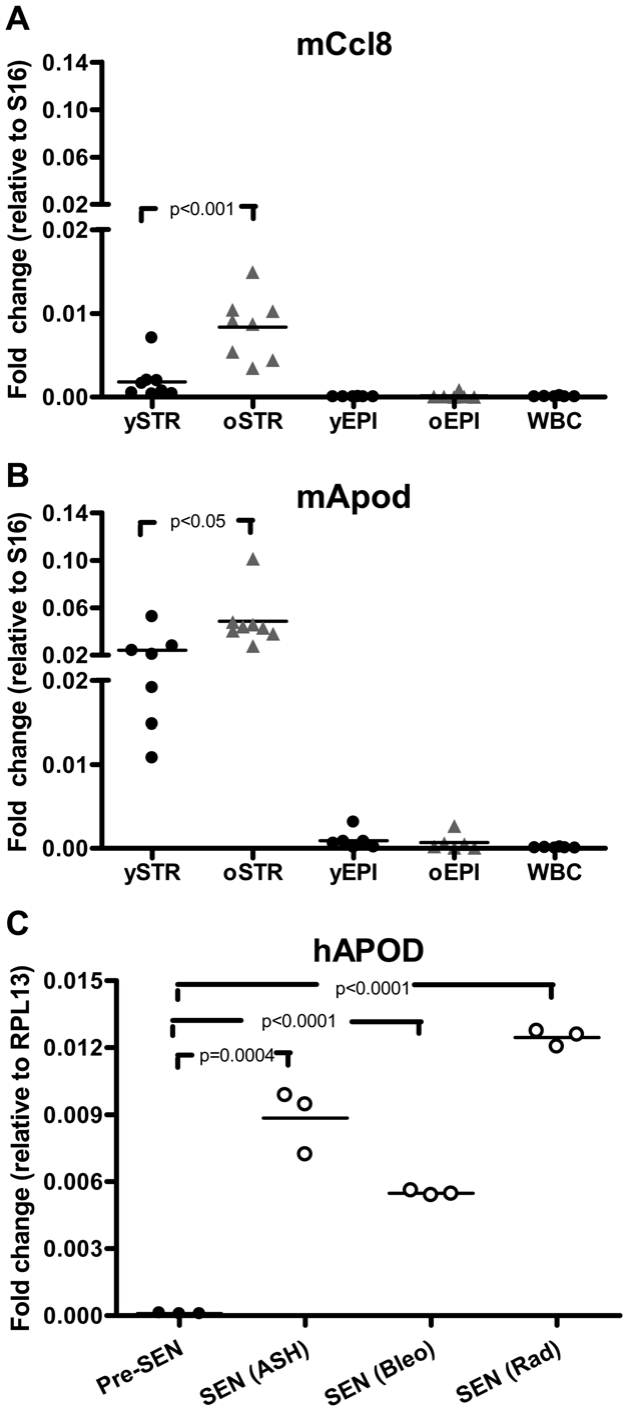
Expression of Ccl8 and Apod with aging, senescence and cell type. (**A,B**) Confirmation of stromal age-related changes in gene expression by qRT-PCR. RNAs were reverse transcribed and amplified using qRT-PCR with primers specific for *Ccl8* and *Apod*. RNAs analyzed: microdissected glandular-adjacent stroma (STR) and epithelium (EPI) from dorsal (n = 4) and anterior (n = 4) prostate lobes from C57BL/6 young (n = 12) and old (n = 12) mice used in microarray analyses. White blood cells (WBC) were isolated from young and old C57BL/6 mice (n = 6). Note the higher expression of *Ccl8* and *Apod* in the microdissected old stroma (Old STR) compared to young stroma (young STR). Also notice the low abundance in transcript levels of these two genes in microdissected young and old epithelium (Young EPI, Old EPI, respectively) and in white-blood cells (WBC). (**C**) Human pre-senescent and senescent prostate PSC27 fibroblasts. Pre-SEN: pre-senescent cells; SEN (ASH) cells induced to senesce by H_2_O_2_ exposure; SEN (Bleo) cells induced to senesce by bleomycin exposure; SEN (Rad) cells induced to senesce by radiation exposure; RPL13 transcript expression levels were used to normalize the human qRT-PCR data.

We next compared transcript abundance levels in the epithelial and stromal cell compartments and identified 378 and 282 genes to be differentially expressed with aging in the stroma and epithelial samples respectively, as determined by a Student's T-test analysis (p<0.05) (**Figure 2C and 2D**). To verify the aging-induced gene expression alterations in prostate stroma, we performed an additional microarray experiment from laser captured microdissected adjacent stroma from an independent set of 4 month-old (n = 12) and 24 month-old (n = 12) C57BL/6 mice and used a more comprehensive microarray platform comprised of oligonucleotides complementary to ∼40,000 genes. Using the same t-test cutoff (p<0.05), 718 transcripts were increased and 541 transcripts decreased in aged versus young prostate stroma (**Supporting information Figure S2**). A significant correlation coefficient of 0.22 (p<0.0001) between the two distinct microarray experiments was determined using the scored T-test for the most differentially expressed genes in both platforms (p<0.05). Experiments using qRT-PCR as an independent measurement confirmed that chemokine (C-C motif) ligand 8 (*Ccl8*) and apolipoprotein D (*Apod*) were increased in aged prostate stroma (**Figure 3A and 3B**, respectively). We also determined that *Apod* and *Ccl8* are expressed at very low levels in white blood cell isolates and in microdissected epithelium relative to stroma, and no differences were seen between young and old epithelium. Several genes were identified with lower expression in aged relative to young prostate stroma including transcripts encoding extracellular matrix proteins *Col1a1*, *Col1a2*, *Col3a1*, among others. We confirmed lower expression levels of these collagen genes by qRT-PCR (see below).

**Figure 4:**
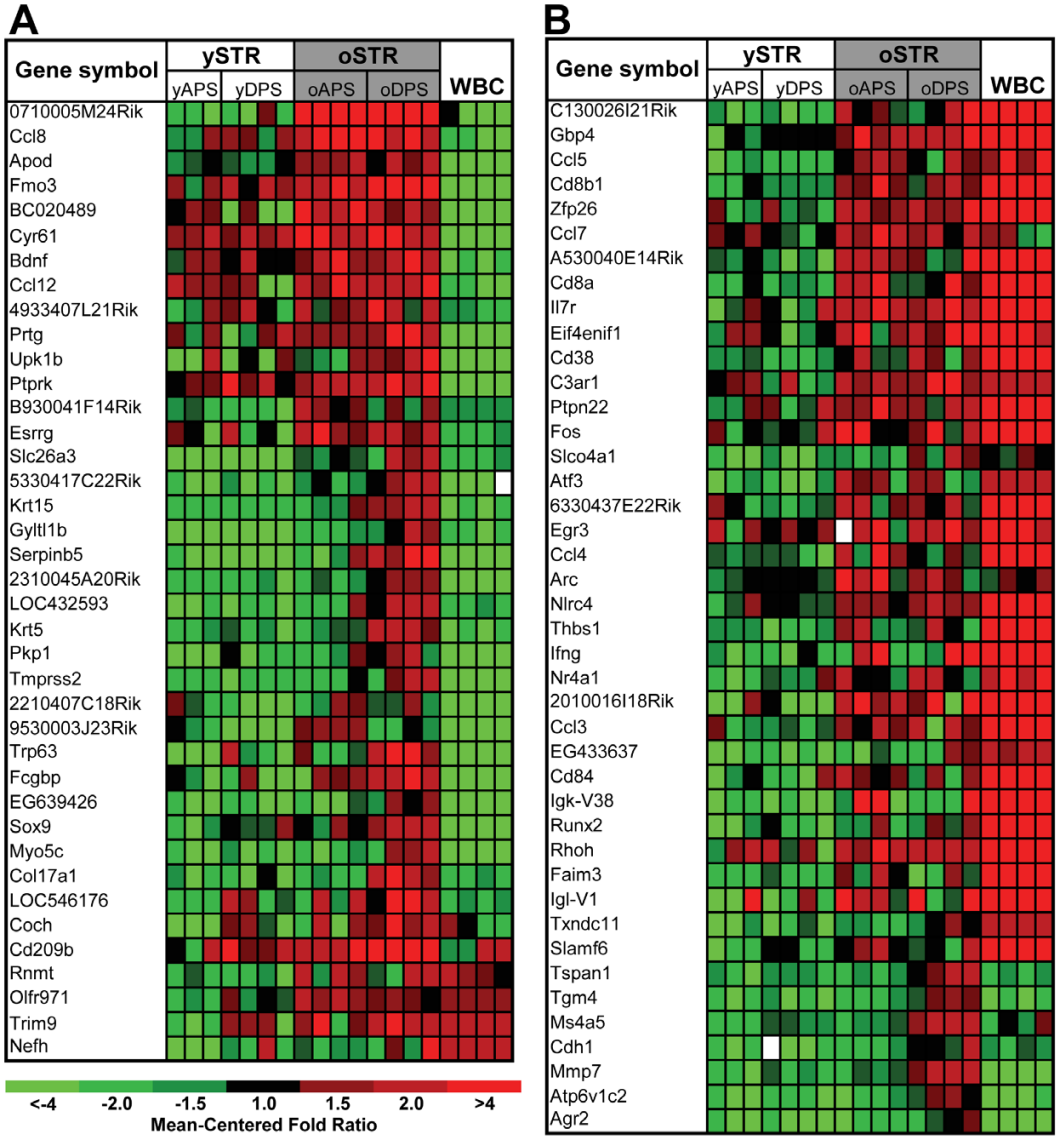
Age and inflammatory cell associated gene expression changes in the mouse prostate stroma. **A**) Intrinsic smooth-muscle/fibroblastic stroma transcriptional profile. Genes whose signal intensity in the white blood cells (WBC) was higher than 800 intensity units were removed and listed in panel B. Note the high expression of genes in the old stroma (oSTR) compared to young stroma (ySTR) and white blood cells (WBC). **B**) Genes significantly up-regulated in the aged stroma that were also expressed in white-blood cells with signal intensity levels higher than 800 intensity units (in WBC). Heat map colors reflect fold ratio values between sample and reference pool and mean-centered across samples. Columns represent biological replicates from dorsal and anterior microdissected cells for each age group and white-blood cells. Rows represent individual genes. Values shown in red are relatively larger than the overall mean; values shown in green are relatively smaller than the overall mean. Gene lists represent the most significant up-regulated transcripts in old stroma compared to young stroma by unpaired, two-sample t-test analysis (p<0.05) with fold changes higher than 2.2. APS = anterior prostate stroma; DPS = dorsal prostate stroma; WBC = white blood cells.

Since histological analysis demonstrated that the aged prostate contains a higher number of inflammatory cells, we were concerned that a component of the aged prostate stroma expression profile could reflect transcripts derived from infiltrating leukocytes. We generated expression profiles from purified white blood cells from C57BL/6 mice (WBC) and compared the expression levels of each age-associated stromal gene with abundance levels in the WBC preparation (**Figure 4**). Considering the most significant up- and down-regulated genes (p<0.05) in the aging data set we found that 59% of the genes determined to be altered in aging stroma overlap with genes expressed in the white blood cell profile. These results suggest that a large component of the age-associated gene expression changes may reflect infiltrating cells (**Figure 4B**), but that substantial changes are also intrinsic to the aging process of the smooth-muscle/fibroblastic stroma and not from differences in numbers of infiltrating leukocytes (**Figure 4A** and **Table 1** **and** **Table 2**). To further evaluate this concept, we examined the prostate glands of young and aged mice of the ICR/SCID strain housed in a barrier facility. In the absence of infiltrating lymphocytes and neutrophils in the prostates of these mice, we confirmed that age-associated changes in smooth muscle cell histology and aging-associated gene expression occurred (e.g. 2-fold increase in *Apod* expression; p<0.05) (**Supporting Information Figure S3**). However, these animals were not formally isolated in a pathogen-free environment, and more rigorous attention to sterility and the elimination of other immune-cell components will be required to evaluate the complex interactions between environment and intrinsic aging.

**Table 1:** Genes with higher expression in the mouse prostate stroma from old (24 months) compared to young (4 months) C57BL/6 mice.

Gene symbol	Gene name (abbreviated)	GenBank accession	Fold change (old/young)
Ralyl	RALY RNA binding protein-like	NM_178631	+35.9
Serpinb5	serine peptidase inhibitor, clade B, member 5	NM_009257	+9.0
Trp63	transformation related protein 63	NM_011641	+7.2
Slc26a3	solute carrier family 26, member 3	BC037066	+6.9
Apol9a	Apolipoprotein L 9a	AK050122	+6.5
Gm11538	predicted gene 11538	AK078606	+6.1
5330417C22Rik	RIKEN cDNA 5330417C22 gene	BC051424	+6.1
Fcgbp	Fc fragment of IgG binding protein	NM_176924	+5.9
Ccl8	chemokine (C-C motif) ligand 8	NM_021443	+4.6
Trim9	tripartite motif protein 9	AK163123	+4.6
Col17a1	procollagen, type XVII, alpha 1	NM_007732	+4.4
Coch	coagulation factor C homolog	NM_007728	+4.2
Ccl12	chemokine (C-C motif) ligand 12	NM_011331	+4.1
Gyltl1b	glycosyltransferase-like 1B	NM_172670	+3.9
Nefh	neurofilament, heavy polypeptide	NM_010904	+3.9
Krt15	keratin 15	NM_008469	+3.8
Fmo3	flavin containing monooxygenase 3	NM_008030	+3.7
Esrrg	estrogen-related receptor gamma	NM_011935	+3.7
Upk1b	uroplakin 1B	NM_178924	+3.6
Myo5c	myosin VC	BC003985	+3.4
9530003J23Rik	RIKEN cDNA 9530003J23 gene	NM_029906	+3.2
Pkp1	plakophilin 1	NM_019645	+3.1
LOC546176	Similar to Spindlin-like protein 2 (SPIN-2)	XM_620811	+3.0
Tmprss2	transmembrane protease, serine 2	NM_015775	+3.0
2210407C18Rik	RIKEN cDNA 2210407C18 gene	NM_144544	+3.0
EG639426	similar to Tetratricopeptide repeat protein 6	XR_003227	+2.9
Rnmt	RNA (guanine-7-) methyltransferase	AK084393	+2.9
Krt5	keratin 5	NM_027011	+2.8
Ptprk	protein tyrosine phosphatase, receptor type, K	AK078614	+2.6
Cd209b	CD209b antigen	NM_026972	+2.6
Sel1l3	sel-1 suppressor of lin-12-like 3	NM_172710	+2.6
Sox9	SRY-box containing gene 9	NM_011448	+2.6
Bdnf	brain derived neurotrophic factor	NM_007540	+2.4
4933407L21Rik	RIKEN cDNA 4933407L21 gene	AK016730	+2.4
B930041F14Rik	RIKEN cDNA B930041F14 gene	NM_178699	+2.4
Prtg	protogenin homolog	AK036172	+2.3
Apod	apolipoprotein D	NM_007470	+2.3
Olfr971	olfactory receptor 971	NM_146614	+2.3
Cyr61	cysteine rich protein 61	NM_010516	+2.3
Npy2r	neuropeptide Y receptor Y2	NM_008731	+2.2
Grhl1	grainyhead-like 1	NM_145890	+2.2
Ric3	resistance to inhibitors of cholinesterase 3	NM_001038624	+2.2
5031410I06Rik	RIKEN cDNA 5031410I06 gene	NM_207657	+2.2
Pcdhga1	protocadherin gamma subfamily A, 1	NM_033584	+2.2
Pigr	polymeric immunoglobulin receptor	NM_011082	+2.2
Cpne5	copine V	NM_153166	+2.2
Cytl1	cytokine like 1	BC063103	+2.2
Slc6a7	solute carrier family 6, member 7	NM_201353	+2.2
Dsp	desmoplakin	AK077574	+2.2
Gjb3	gap junction membrane channel protein beta 3	NM_008126	+2.1
4930591A17Rik	RIKEN cDNA 4930591A17 gene	NM_026596	+2.1
Edg4	endothelial diff. lysophosphatidic acid GPCR 4	NM_020028	+2.1
Faim2	Fas apoptotic inhibitory molecule 2	NM_028224	+2.1

Microarray analysis of gene expression in microdissected mouse prostate stroma from old (24 month) compared to young (4 month) C57BL/6 mice. Unique genes (n = 53) with significantly increased transcript levels between aged and young samples (p<0.05) and whose expression levels were low in white blood cells. Fold changes were calculated from the averages across multiple anterior and dorsal prostate samples in each group. Positive values indicate an increase in gene expression in aged prostate as compared to young.

**Table 2:** Genes with lower in expression in the mouse prostate stroma from old (24 months) compared to young (4 months) C57BL/6 mice.

Gene symbol	Gene name (abbreviated)	GenBank accession	Fold change (old/young)
Lrat	lecithin-retinol acyltransferase	NM_023624	−4.4
Uxs1	UDP-glucuronate decarboxylase 1	AK078575	−4.2
A930025H08Rik	RIKEN cDNA A930025H08 gene	AK020889	−3.8
Adamts19	ADAM with thrombospondin type 1 motif, 19	NM_175506	−3.7
Nrxn1	neurexin I	NM_020252	−3.6
6530401D17Rik	RIKEN cDNA 6530401D17 gene	NM_029541	−3.3
2900006F19Rik	RIKEN cDNA 2900006F19 gene	NM_028387	−3.2
D930015E06Rik	RIKEN cDNA D930015E06 gene	AK153996	−3.1
Prnd	prion protein dublet	NM_023043	−3.1
Atp1b3	ATPase, Na+/K+ transporting, beta 3	AK078587	−3.1
5730507A11Rik	RIKEN cDNA 5730507A11 gene	AK020490	−3.0
Abhd2	abhydrolase domain containing 2	NM_018811	−3.0
Sox11	SRY-box containing gene 11	AF009414	−3.0
C630004L07	hypothetical protein C630004L07	AK049864	−2.9
Nfrkb	nuclear factor related to kappa B binding	AK036381	−2.8
Zfp715	zinc finger protein 715	AK011730	−2.7
Grik4	glutamate receptor, ionotropic, kainate 4	NM_175481	−2.7
EG621699	similar to 40S ribosomal protein S7 (S8)	AK048878	−2.7
Fzd3	frizzled homolog 3	NM_021458	−2.6
4833446K15Rik	RIKEN cDNA 4833446K15 gene	XM_894542	−2.6
Gdap1	ganglioside-induced diff. associated-protein 1	NM_010267	−2.6
Calcb	calcitonin-related polypeptide, beta	NM_054084	−2.5
Pde11a	phosphodiesterase 11A	AK050924	−2.5
Cacna1g	calcium channel, volt. dep., T type, alpha 1G	NM_009783	−2.4
Fbxo7	F-box only protein 7	BC059894	−2.4
Ccdc106	coiled-coil domain containing 106	NM_146178	−2.4
Col1a1	procollagen, type I, alpha 1	NM_007742	−2.4
Pcdhb4	protocadherin beta 4	NM_053129	−2.3
BC068157	cDNA sequence BC068157	NM_207203	−2.3
Bub1	budding uninhibited by benzimidazoles 1	NM_009772	−2.2
Tomm70a	translocase of outer mitoc. membrane 70 A	AK122356	−2.2
LOC665113	similar to Traf2 and NCK-interacting kinase	XM_976833	−2.2
Ppp2r2b	protein phosphatase 2, regulatory subunit B	NM_028392	−2.1
Bub3	budding uninhibited by benzimidazoles 3	AK083742	−2.1
Kif4	kinesin family member 4	NM_008446	−2.1
Pcdhb7	protocadherin beta 7	NM_053132	−2.1
Fgf12	fibroblast growth factor 12	NM_183064	−2.1
Plxna3	plexin A3	AK049319	−2.1
Tmem121	transmembrane protein 121	NM_153776	−2.1
Epb4.1l3	erythrocyte protein band 4.1-like 3	AK086340	−2.1

Microarray analysis of gene expression in microdissected mouse prostate stroma from old (24 months) compared to young (4 months) C57BL/6 mice. Unique genes (n = 40) with significantly lower expression in aged compared to young samples (p<0.05) and whose expression levels were also low in white blood cells. Fold changes were calculated from the averages across multiple anterior and dorsal prostate samples in each group. Negative values indicate a decrease in gene expression in aged prostate as compared to young.

**Correlations between** ***in vivo*** **aging and** ***in vitro*** **senescence**

Although the molecular process of replicative senescence is intimately associated with features of aging, it has been challenging to directly determine whether senescent cellular phenotypes normally accumulate with aging in numbers sufficient to influence pathological processes *in vivo*. We selected several genes known to be associated with *in vitro* senescence and compared their transcript levels between young and aged prostate stroma. The first cohort we evaluated were those comprising a senescence-associated secretory phenotype (SASP) shown to directly influence epithelial cell growth [11]. Unexpectedly, none of the senescence associated candidate factors we evaluated, *Hgf*, *Ctgf*, *Fgf7*, *Cxcl12*, *Areg*, *Il6*, *Il1a*, and *Cxcl1* were up-regulated in the aged mouse prostate stroma (see **Supporting information Figure S4**, for genes whose transcripts were detectable in microdissected stroma). However, transcripts we found to be elevated in aged stroma *in vivo*, such as *Apod* and *Ccl8*, were also up-regulated in primary human prostate fibroblasts that were induced to senesce *in vitro* (**Figure 3C**).

Employing a more systematic approach, we compared the transcriptional profiles of aged murine stroma measured in this study (considering a false discovery rate of <25% and including those transcripts also found in leukocytes), with previously determined transcriptional profiles of human prostate fibroblasts induced to senesce by different means (H_2_O_2_, Bleomycin, replicative senescence, overexpression of p16 and overexpression of oncogenic RAS [11] and unpublished data). Of 264 genes significantly altered in aged mouse stroma, 37 genes were also significantly altered in at least one senescence profile (FDR<25%) (**Supporting information Figure S5**). Genes involved in the NF-κB pathway, such as *STAT1* and *TLR1*; cell proliferation/apoptosis, such as *IER3*; *EHF*; *LRPAP1* and inflammation such as *CCL7*; *CXCL16*; *B2M*; *IL7R* and *LGALS3* were among the genes whose transcripts were increased in the context of both *in vivo* aging and *in vitro* senescence. The changes within these gene groups are in agreement with the age-enriched biological functions identified by pathway-based analyses described below.

### Prostate aging influences specific molecular pathways

To determine if the age-induced gene expression alterations comprised specific biological programs, we used gene set enrichment analysis (GSEA) to determine the statistical associations of predetermined gene cohorts. We used the Gene Ontology (GO) set (C5) and curated gene sets (C2) which included canonical pathways, and a senescence-associated gene list generated based on gene expression changes quantitated in human prostate fibroblasts [11]. We found 164 GO gene sets to be significantly enriched in the aged stroma (FDR<25%.). These included enrichment of ‘Inflammatory Response’ and ‘Cytokine/Chemokine Activity’ categories (NOM P-value <0.05; FDR <2%). Genes involved in the ‘NF-κB Cascade’ were also enriched in the aged stroma (NOM P-value <0.05; FDR <25%), consistent with prior studies linking intracellular regulation of immune responses in both aging and age-related diseases [32]. GSEA also determined that the category of ‘collagen binding and collagen genes’ was significantly enriched for transcripts downregulated in aged stroma (NOM p-value<0.05 FDR <25%). Additionally, using the database of curated gene sets, we found that gene sets derived from aged mouse neocortex, cerebellum, kidney and retina were enriched as well as the senescence-associated gene list derived from senescent prostate fibroblasts (NOM p-value <0.05 FDR <25%). GenMAPP 2.1 was used to visualize the age-associated expression changes in pathways found significant by GSEA. A representation of the Cytokine and Inflammatory response pathway is shown in **Supporting Information Figure S6**.

### Effects of aging on prostate extracellular matrix components and tissue architecture

**Figure 5:**
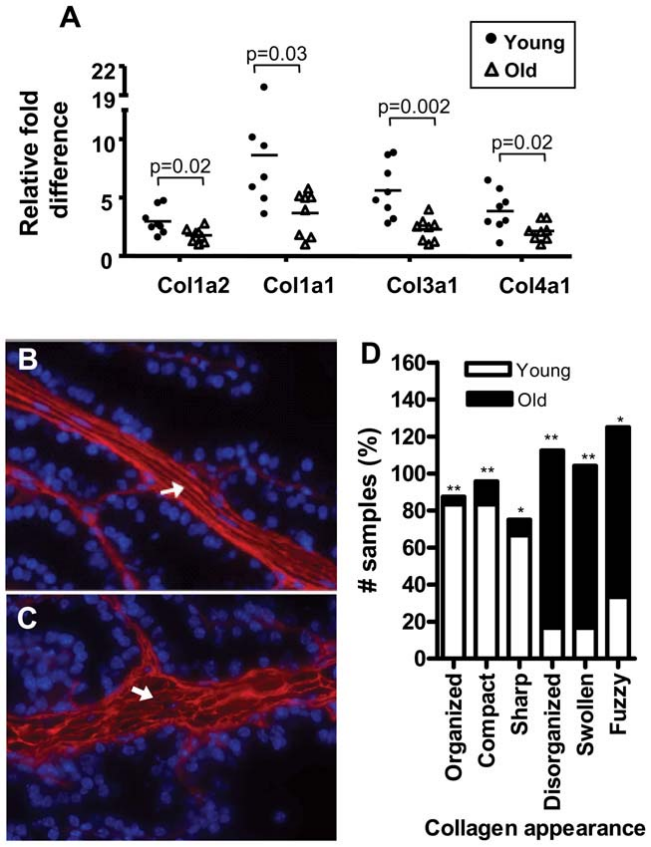
Age-related alterations in collagen expression and structure. **A**) Analysis of Col1a2, Col1a1, Col3a1 and Col4a1 transcripts by qRT-PCR from microdissected young (n = 8; 4-month-old) and old (n = 8; 24-month-old) anterior prostate stroma. Circle: young; Triangle: old. Ribosomal S16 transcript expression levels were used to normalize qRT-PCR data. Normalized results are expressed relative to the lowest expression value for each gene tested. **B–D**) Qualitative and quantitative confocal microscopy analysis for the appearance of collagen fibers in young and old mouse prostate. Thirty micrometer sections of anterior prostate lobes were stained by immunofluorescence with Collagen Type I antibody and were evaluated by confocal microscopy. Six scoring criteria were used to quantify the differences in collagen fiber appearance (organized, compact, sharp, disorganized, swollen and fuzzy collagen fibers). Five young and five old anterior prostates from independent mice were used and 4 images were taken from each sample **p<0.001 and *p<0.005. **B, C**) Representative images of Collagen Type I immunofluorescent stain of frozen sections from 4- (B) and 24-month (C) old mice (Magnification: 40×). Note the coarse and fragmented appearance and less regular distribution of collagen fibers in old prostates compared to the fine collagen fibers and highly organized network in the young prostate (arrows).

The transcript profiling studies determined that genes encoding structural extracellular matrix components were expressed at lower levels in aged relative to young prostate stroma. These included several collagen-encoding genes; collagen I α1 and α2 subunits, and collagen III α1 subunit. We confirmed significantly lower transcript levels of *Col1a1*, *Col1a2*, *Col3a1* and *Col4a1* in aged stroma by qRT-PCR (**Figure 5A**). To further investigate these findings, we used fluorescence and ultrastructural microscopic analysis to determine the relationship between collagen structure and aging. Examination of the extracellular matrix (ECM) surrounding prostate epithelial cells by immunofluorescent staining for Type I collagen and picrosirius red (a selective staining agent for collagen; data not shown) demonstrated that the majority of the stroma around the prostatic ductal structure is fibrillar collagen (**Figure 5B and 5C**). Interestingly, although immunofluorescence detection for Type-I collagen did not show substantial differences in the protein abundance between young and old prostate tissue, it revealed a disorganized collagen matrix network with a coarse, fragmented, and less ordered distribution of the collagen fibrils in prostates from old animals compared to the fine collagen fibrils and highly organized network in prostates from young animals (**Figure 5C and 5B**, respectively). In order to rule out that the disorganization of the collagen fibers was due to mechanical damaging during sectioning, 30 µm sections of the anterior prostate lobe were stained by immunofluorescence with antibodies recognizing Type I collagen and were evaluated by confocal microscopy in order to obtain a stack of images inside the intact tissue. Six scoring criteria (see materials and methods section) were used to quantify the differences in collagen fiber appearance (organized, compact, sharp, disorganized, swollen and fuzzy collagen fibers). Collagen fibril appearance was significantly different between young and old prostates, with more than 70% of the aged prostates evaluated having disorganized, swollen and fuzzy collagen fibers (p<0.05) compared to the organized, compact and sharp collagen fibril appearance from young mice (p<0.005) (**Figure 5D**). Similar alterations were observed in sections from the dorsal, lateral and ventral lobes; however, to quantify the observations, the more abundant stroma layer in the anterior lobe was chosen to facilitate the scoring.

**Figure 6:**
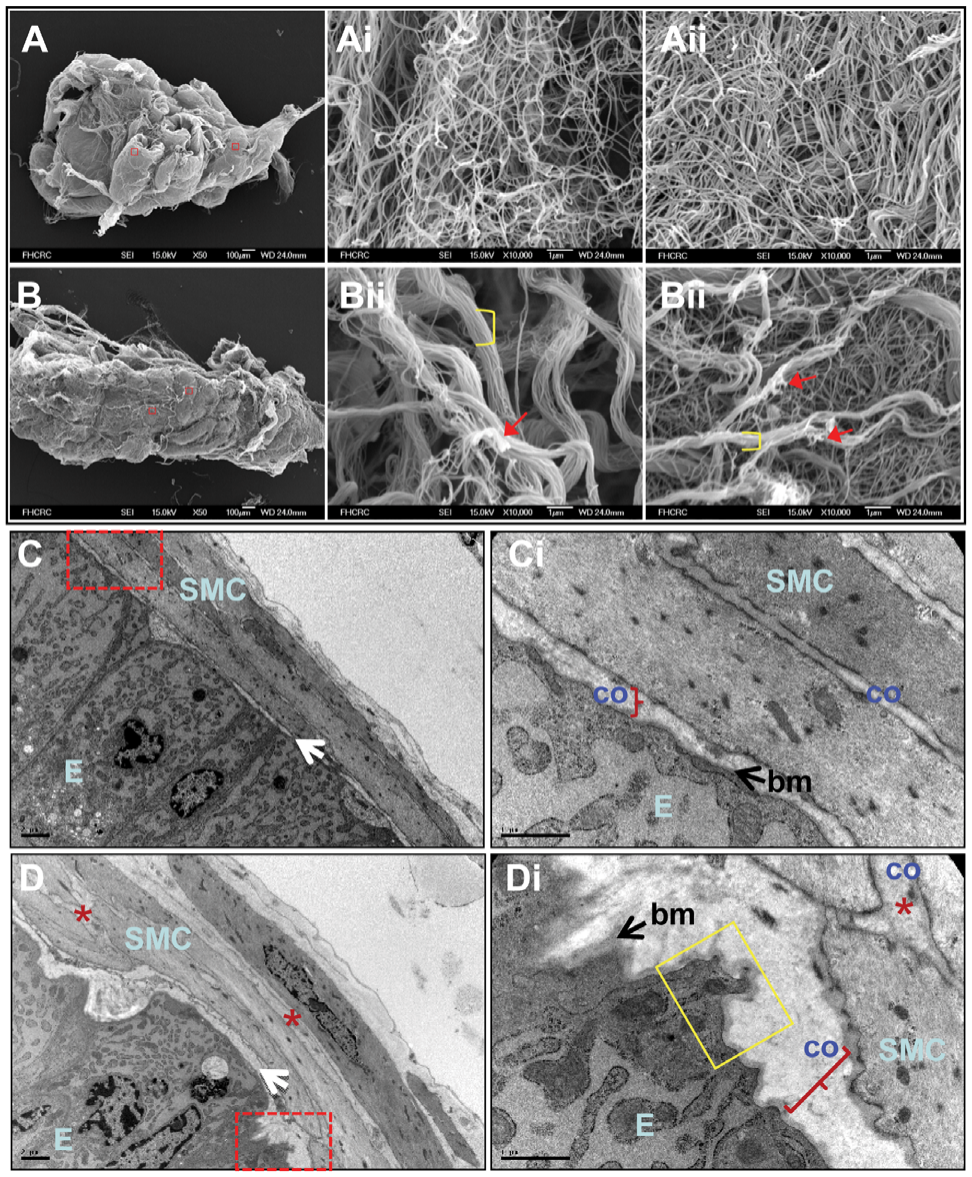
Ultrastructure of the young and aged mouse prostate. **A and B**) Scanning electron microscopy of acellular preparations from young (A) and old (B) anterior prostate. General view of the anterior prostate from young (A) and old (B) mice. **Ai–Bii**) Representative images of high power fields from young (Ai and Aii) and old (Bi and Bii) anterior prostate. Note the collagen meshwork of loosely woven fibrils with an intact structure of distinct collagen bundles in the young prostate (Ai and Aii) compared to the adhered collagen bundles (brackets) and fragmentation of collagen fibrils (arrows) in aged prostate (Bi and Bii). This phenotype was observed in all analyzed samples and in different selected random field (young n = 5 and old n = 5). **C and D**) Transmission electron microscopy of cross sections from young (C, Ci) and old (D, Di) normal mouse prostate. C and D) general view of the glandular-adjacent stroma in proximity to the prostatic epithelium (E). Red dashed square: region presented in Ci and Di. Ci and Di) Detail of the epithelial-stromal interface in young (Ci) and old (Di) prostates with a thick basement membrane (bm). A thick layer of collagen fibrils (“co” and brackets) are distributed at the epithelium base and interspersed with smooth muscle cells (SMC). Yellow square: detail of an epithelial cell (E) with cytoplasmic expansions compressing the basement membrane. **E** indicates luminal epithelial; **SMC**, smooth muscle cells; **bm** and arrow, basement membrane; **co**: and brackets: collagen fibrils. These sections are representative of sections obtained from 4 prostates for each age group.

To investigate in greater detail the structural alterations of the collagen network in the aged prostate, we performed scanning electron microscopy using prostates from young and old animals. To visualize the three-dimensional organization of the collagenous stroma, samples were treated with serial washes of 10% NaOH solution to remove all cellular elements [33], [34]. The acellular preparations (**Figures 6A and 6B**) showed that a smooth and grossly homogeneous fibrous network lines the empty acinar space. Due to difficulties imaging the internal ducts of the acini it was not possible to assess whether this surface exhibits differences between young and old prostates. However, on the outside of the ducts, a sponge-like organization was apparent. The young prostates displayed a meshwork of loosely woven fibrils comprised of distinct collagen bundles, while in aged mice collagen bundles were adherent or joined to each other (**Figures 6A** **i–Aii** and **6Bi–Bii**, respectively). These observations were similar to those seen by immunofluorescence staining for Collagen Type I (**Figure 5B and 5C**). Collectively, the collagenous stroma in the aged mouse prostate is characterized by a disorganized and disrupted collagen matrix.

To further characterize the prostate at the ultrastructural level, we performed transmission electron microscopy (TEM) analysis of young and old mouse prostates (**Figure 6C and 6D**, respectively). In agreement with the immunofluorescence analysis for Collagen Type I, we found that despite the lower levels of procollagen I alpha-1 mRNA in the aged animals, they also exhibited a dramatic increase in collagen fibers in the stroma as determined by TEM (compared **Figure 6C** **i** and **6Di**, brace). Additionally, since we demonstrated that the basement-membrane-codifying pro-collagen Col4a1 mRNA was down-regulated with aging (**Figure 5A**), we then analyzed the organization of the basement membrane by TEM in young and aged prostates. Although we did not find any obvious structural alterations or disruptions in the basement membrane between age groups, we did observe epithelial cytoplasmic projections extending towards the extracellular matrix in the aged prostate (**Figure 6D** **i**, yellow square), suggesting that the aged basement membrane is less rigid, thus allowing these epithelial cells to compress it towards the extracellular matrix. Consistent with these findings, similar ultra-structural phenotypes have recently been observed in the aged Mongolian gerbil ventral prostate [35]. In addition to the collagen changes, we also sought to determine whether the cells (mainly smooth muscle cells) within the altered extracellular matrix presented proper organization. In aged prostates, the smooth muscle cells presented a less consistent orientation within the stroma and did not have a continuous parallel arrangement as observed in young prostate (**Figure 6D–D** **i**, asterisk, and **6C–Ci**, respectively).

### Effects of normal aging on prostate inflammation

**Figure 7:**
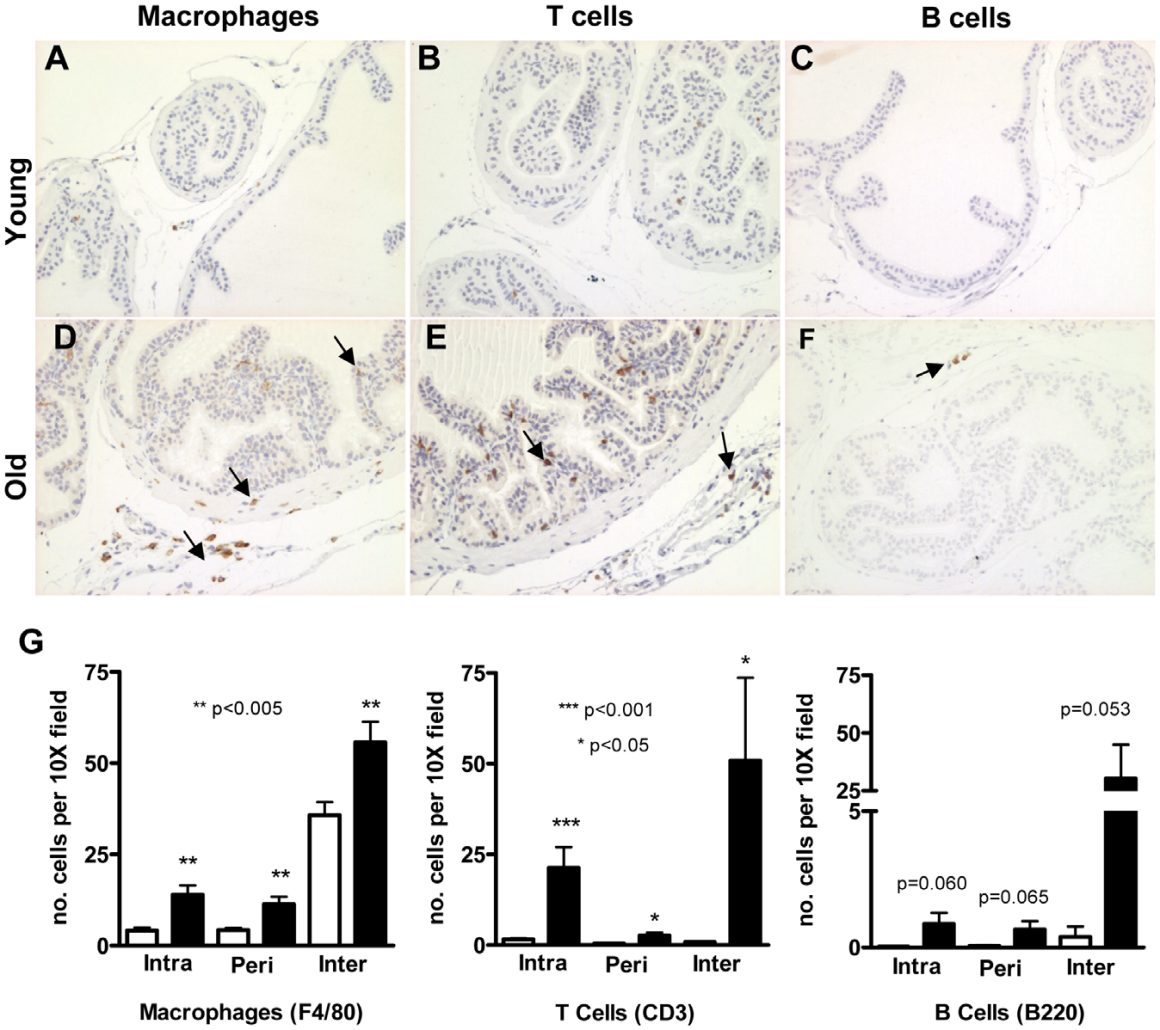
Prevalence of inflammatory cells in prostates from aged mice. **A–F**) Immunohistochemical analysis of 4 µM paraffin sections from anterior prostate of young (A–C) and old (D–F) mice. Sections were stained with anti-F4/80 (A and D) anti-CD3 (B and E) and anti-B220 (C and F), which recognize macrophages, T cell and B cells, respectively. IHC demonstrated a high number of inflammatory cells within the aged prostate tissue. **G**) The number of cells positive for each immune-cell marker were determine by the number of cells/10X field on each lobe by blinded section analysis from young (4 month-old; n = 10) and old (24 month-old; n = 13) prostate sections. Inflammatory infiltrates were divided into three different categories: intraglandular infiltrates (inflammatory cells in contact with the glandular luminal epithelium); periglandular stromal infiltrates (inflammatory cells in contact with the smooth-muscle/fibroblastic cellular stroma); and interglandular infiltrates (inflammatory cells in the interglandular space). Data are mean ± standard error for all lobes combined. ***p<0.001; **p<0.005 and *p<0.05.

The gene expression changes measured in the stroma of aged mouse prostate was indicative of a pro-inflammatory environment. Increased expression of inflammation-associated genes suggested that the stroma cells in the older animals were both responding to, and producing inflammatory signals. We next evaluated prostate tissues from aged (n = 10) and young (n = 10) animals for several markers that specify inflammatory cell types: F4/80 (macrophages), CD3 (T cells) and B220 (B cells) (**Figure 7A–F**), and determined that the number of B cells, T cells and macrophages were significantly increased in the aged prostate (B cells, p = 0.049; T cells, p = 0.003; Macrophages p<0.001; **Figure 7G**). We then evaluated the localization of these immune cells and determined the number of cells positive for each immune cell marker. Inflammatory infiltrates were divided into three different categories: periglandular stromal infiltrates (inflammatory cells in contact with the smooth-muscle/fibroblastic cellular stroma); intraglandular infiltrates (inflammatory cells in contact with the glandular luminal epithelium); and interglandular infiltrates (inflammatory cells between glands). The number of intraglandular, periglandular, and interglandular macrophages and T cells were significantly higher in aged prostates compared to young prostates (p<0.05) (**Figure 7G** and **Supporting information Figure S7**). There were low numbers of B cells in contact with the epithelium and smooth-muscle cells (range from 1–16 cells per 10x field) and B cells were absent in most prostatic regions in both young and aged mice. However, in the interglandular space, B cells were present in a significantly higher number in the aged prostate compared to young prostates (p = 0.053) (**Figure 7G**). Although we did not stain the prostate gland for NK cells, higher levels of transcripts encoding NK specific markers (Nkr-PC1, Nkr-P1A and Cd49b) were found in the prostate tissue of the aged mice (p<0.05; data not shown). These observations, along with the increased expression of immune-specific genes (especially chemokines and immunoglobulin genes) are consistent with the increased infiltration of lymphocytes and macrophages in the aged mouse prostate.

The elevated numbers of inflammatory cells in the aged prostate prompted us to investigate the potential reasons for this finding. By H&E staining of prostate tissues from each age group, we were able to discard the possibility of an inflammatory response due to bacterial infections, since neither obvious bacteria nor associated neutrophilic infiltrates were present in the mouse prostates. Thus, one reason for the presence of the inflammatory cells in the aged prostate might be a consequence of increased levels of chemokines/cytokines originating from the aged smooth-muscle fibroblastic stroma. However, we cannot rule out the possibility that the transcripts encoding these cytokines, at least *Ccl7* and *Ccl5,* are derived directly from the infiltrating inflammatory cells in the stromal samples (**Figure 4B**). To date we have not been able to demonstrate whether the increased levels of chemokines, such as *Ccl8* in aged prostate originate exclusively from smooth-muscle/fibroblastic cellular compartment or includes a contribution from infiltrating cells. However, the fact that *Ccl8* and *Apod* were not expressed in the peripheral leukocyte populations isolated from young and old C57BL/6 mice, suggests that these alterations are likely to be intrinsic to the smooth muscle cells and fibroblasts.

## Discussion

Studies in humans and other mammals have shown that frequently encountered disorders of prostate growth, classified as benign prostatic hyperplasia (BPH) and prostate carcinoma, are associated with aging [36], [37], [38], [39], [40], [41], [42]. However, there is no established molecular explanation for the profound age-dependent increase in these diseases. Thus, our intent was to obtain an unbiased view of the histological and molecular changes in the microenvironment of the prostate gland that accompany advancing age, with a view toward defining those features capable of influencing prostatic pathology. To this end, we used an inbred mouse strain to isolate intrinsic aging-associated features from effects attributable to genetic variation, diet, or external environment.

Histological analyses of the prostate did not show quantifiable differences in the glandular compartment between young and old mice; however changes in the non-epithelial microenvironment were clearly evident with increased numbers of inflammatory infiltrates and a collapsed appearance of the smooth muscle cells present within the glandular-adjacent stroma. This observation is in agreement with studies in aged human skin where reduced fibroblast spreading has been proposed to be indicative of diminished mechanical tension due to a lack of direct association of the fibroblasts with age-related fragmented collagen fibrils [43], [44]. These alterations in mechanical tension and cell shape have been suggested to be critical determinants of cellular function [45], [46], implying changes in gene expression. Thus, we anticipated that smooth-muscle cells and fibroblasts in the aged prostate might exhibit alterations at the molecular level, reflecting age-related structural modifications, a response to the infiltration of inflammatory cells, or stemming from other intrinsic aging-related alterations.

In order to identify specific and consistent aging-associated alterations in gene expression in the glandular adjacent stroma, a region primarily comprised of smooth muscle cells, we used laser capture microdissection and full-genome transcript profiling to quantitate transcript abundance levels. In a comparative analysis between the microdissected stroma and epithelial cells we were able to show that little overlap exists in age-related gene expression between these two compartments, suggesting that aging phenotypes are not uniform in nature. Consistent with this concept, several studies have shown that patterns of gene expression associated with aging or senescence is strongly influenced by tissue type and/or cell lineage [47], [48], [49]. Analyses of gene networks encompassing the alterations observed between young and aged prostate stroma demonstrated a significant enrichment for inflammation pathways, the NF-κB program, and collagens, among others. Consistent with these results, analyses of the aged rat prostate [37], [38] as well as global studies using different tissues and species including mice and humans [50], identified greater expression of inflammatory and stress-response genes, along with a decrease in extracellular matrix components with age [50], [51], [52]. However, these studies could not determine which cellular compartment contributed to the expression changes, as they were performed using whole tissues. In contrast, our study demonstrated that genes up-regulated in the aged prostate, such as *Apod* and *Ccl8* were specifically associated with the stroma compartment and not the aged epithelium.

Using Gene Set Enrichment Analysis we determined that genes altered in human prostatic senescent fibroblasts are enriched in aged stroma, especially those involved in inflammatory responses. However, we also found that many of the key factors comprising the senescence-associated secretory program (SASP) [11] were not altered in the aged mouse stroma. This observation suggests that senescent cells do not accumulate in sufficient numbers with advancing age to contribute substantially to the expression profile of the composite transcriptome ascertained from the stroma. Alternatively, the smooth muscle cell phenotype that dominates the prostate stroma may engage an aging/senescence program distinct from that of fibroblasts.

Considering that inflammatory processes can induce cell stress and that stressed mesenchymal cells can secrete inflammatory chemoattractants [11], [53], it remains to be determined whether inflammatory infiltrates are a cause of, or response to, the molecular alterations exhibited by aged stroma. While the chemokines *Ccl8*, *Ccl12*, *Ccl5* and *Ccl7* found to be elevated in the context of aging may directly promote inflammation, the specific influence of each of these cytokines in the prostate remains to be established. We were able to demonstrate an increase in T cells, macrophages and to a lesser extend B cells in the aged prostate. These cells were not only present in clustered foci in the interglandular regions but were also found infiltrating into the smooth-muscle/fibroblastic stroma as well as the luminal epithelium, and could be effectors of age-related pathologies. A link between genotoxic stress and the activation of the innate immune system through NF-κB has been proposed as a cause of premature aging [32]. Chronic inflammation has emerged as a potential risk factor for carcinomas in many organs such as the liver, colon, bladder, lung and pancreas [54], and there is compelling evidence supporting a role for inflammation in the pathogenesis of prostate cancer [55], [56], [57]. Thus, our observation demonstrating the prostate of aged animals harbors increasing numbers of immune cells suggests that aging correlates with a pro-inflammatory state which in turn may well influence prostate neoplasia.

A striking age-associated finding involved the development of an abundant, highly disorganized and fragmented collagen matrix in the prostate. These collagen alterations may be a consequence of age-associated changes of collagen cross-linking [58], [59], [60] and impairment of its degradation [61] rendering an accumulation of partially degraded fibrils [44]. Of relevance to the gene expression measurements, collagen fragmentation has been shown to promote oxidative stress [44] and could explain the increased expression of stress response genes, such as *Apod* as well as the increase in inflammatory infiltrates. Pertinent to the regulation of collagen in the prostate, androgen deprivation induces marked morphological changes in rat prostatic smooth muscle cells [62], [63] reduces collagen synthesis, and induces a collagen fibrillar reorganization in the rat ventral prostate [64], [65], [66]. On the other hand, 17 beta-estradiol increases the accumulation of collagen in the prostate [67]. It is possible that the hormonal imbalance related to the aging process, together with the potential loss in mechanical tension of the smooth-muscle cells in the aged prostate stroma, as observed by classic histology and transmission electron microscopy, may well affect collagen synthesis and organization.

While the descriptive characteristics of the collagen network are important in our understanding of structural changes with aging, their functional role(s) and biological relevance in prostate pathology remain to be elucidated. In this context, recent evidence supports collagen content, cross-linking, fiber structure, and organization, as key determinants of tumor cell behavior: higher collagen density was shown to increase tumorigenesis, local invasion, and metastasis of mammary epithelial cells, causally linking an increase in stromal collagen to tumor formation and progression [68]. In the human prostate, the collagen tissue network appears to be altered in prostate pathologies (BPH and adenocarcinomas) [34], resembling in part the morphological changes in the collagen matrix observed in the normally aging mouse prostate. These changes at the ultrasctructural level may have implications for prostate growth in normal and pathologic states, though mechanistic cause-effect studies will be required to confirm this hypothesis. In this context, collagen type IV has been shown to enhance the growth of rat ventral prostatic epithelial cells *in vitro* [69], and type I collagen mediates proliferative responses of prostate carcinoma cells [70].

In summary, the studies of the murine prostate microenvironment reported herein demonstrate consistent molecular and structural changes that accompany advancing age. Future studies should be directed toward confirming that these alterations also associate with advancing age in the human prostate gland, and determining which alterations are influenced by genetic variation and which can be modified by environment. Though distinct from the robust senescence program observed *in vitro*, the dysregulated cytokines, stress-response factors, and matrix components identified *in vivo* may causally influence prostate pathologies and should be considered for intervention strategies that could delay or reverse the onset of BPH, LUTS, and prostate cancer.

## Materials and Methods

### Ethics Statement

All animal studies were approved by the Institutional Animal Care and Use Committee at the Fred Hutchinson Cancer Research Center, IR#1671.

### Tissue acquisition and microdissection

Young (4 month-old ) and old (20–24 month-old) C57BL/6 male mice were obtained from the National Institutes of Aging (NIA) Rodent Colony at Harlan Sprague Dawley (Chicago, IL) and cared for in accordance with approved IACUC protocols. For studies of immunocompromised mice, two month-old ICR-SCID male mice were obtained from Taconic and maintained and aged in a barrier facility. Mice in the barrier suite were housed in sterilized microisolator cages using Allentown HEPA-filtered and ventilated racks. Work done in these rooms took place within a HEPA-filtered hood. Animals were sacrificed and prostate tissue dissected when they reached the desired age: 4 months and 13 months for young and old mice respectively. Following shipment, mice were acclimated to a common temperature, day-night cycle, and diet for at least 12 days to minimize environmental differences. Mice from each age group were randomized for the day and time of sacrifice. Following halothane anesthesia, mice were sacrificed by cervical dislocation. Prostates were rapidly excised, immersed in OCT embedding compound (Miles Diagnostics, Elkhart, Ind., USA) and snap-frozen in liquid nitrogen and stored at −80°C.

Frozen sections (7 µM) from young and aged animals were cut from OCT embedded snap-frozen mouse prostate glands into PAP-membrane slides and immediately fixed in 95% ethanol for five minutes, washed in deionized RNase-free water, and stained with Mayer's hematoxylin for 30 seconds, followed by another water wash. The sections were then dehydrated with two five-minute washes in 100% EtOH. Approximately 10,000 glandular-adjacent stroma cells were separately captured from the anterior and dorsal prostate lobes from 17 independent animals of each age group as well as anterior and dorsal prostate luminal epithelium from 5 animals in each age group using the Veritas LCM system (Arcturus Mountain View, CA). Digital photos were taken of tissue sections before, during, and after LCM and assessed independently to confirm the cell type-specificity of the captured cells. To control for individual mouse variability [71], the microdissected stromal samples were combined for total RNA isolation into four pools representing three mice from each aged group and prostatic lobe. Prior to microarray hybridization, cell-type specific purity was verified by qRT-PCR using primers for known stromal (smooth muscle-actin, vimentin) and epithelial (probasin) markers.

### Gene expression analysis by microarray hybridization

All data is MIAME compliant, and the raw data have been deposited in a MIAME compliant database, the Gene Expression Omnibus (GEO) database, as detailed on MGED Society website http://www.mged.org/Workgroups/MIAME/miame.html, and is available at www.ncbi.nlm.nih.gov/geo, under accession GSE21542.

Total RNA from LCM experimental samples were isolated using the Arcturus PicoPure RNA Isolation kit (Molecular Devices, Sunnyvale, CA) incorporating DNAse-treatment using the RNase-Free DNase Set (Qiagen Inc, Valencia, CA.). To provide a reference standard RNA for use on two-color cDNA microarrays, we pooled total RNA isolated from normal adult male Swiss-Webster mice (10% prostate and 30% each testis, liver, and kidney.) Reference RNA was purified using Trizol (Life Technologies, Rockville, MD) following the manufacturer's protocol followed by further purification by RNeasy maxi kit (Qiagen Inc, Valencia, CA) including DNAse treatment using the RNase-Free DNase Set (Qiagen Inc, Valencia, CA.) Total RNA from experimental samples were amplified two rounds and reference total RNA was amplified one round using the Ambion MessageAmp aRNA Kit (Ambion Inc, Austin, TX), according to the manufacturer's specifications. The amplified RNA was used as template for cDNA probe synthesis followed by hybridization to a custom mouse prostate cDNA array (MPEDB array) composed of approximately 8,300 genes expressed in the developing and adult mouse prostate [72], or to a 44K oligonucleotide microarray (Agilent, Inc). Probe synthesis, microarray hybridization and data acquisition were performed as described previously [31], [73]. Spots of poor quality or average intensity levels <300 were removed from further analysis.

To identify genes that varied between young and aged mouse prostate, log2 ratio measurements were statistically analyzed by a Student's T-test analysis (unpaired, two-tailed, unequal variance), and transcripts with p-values <0.05 were considered significantly altered between young and old stroma. Transcriptional profiles of the epithelium and stroma samples from young and old mice were also compared with Principal Components Analysis (PCA) using Bioconductor software [74].

To identify specific biological pathways that exhibit age specific alterations, microarray results were subjected to Gene Set Enrichment Analysis (GSEA) [75]. Genes were preranked based on the T-test score between young and aged mouse prostate. For genes represented by multiple probes, we used the T-test statistic from the probe that had the largest absolute T-test statistic. GSEA was run in preranked mode using the Gene Ontology (GO) set (C5) and curated gene sets (C2) from the Molecular Signatures Database using 1000 permutations to estimate the false discovery rate to assess statistical significance. An FDR of < 25% was considered significant. GenMAPP 2.1 (www.genmapp.org) was used to visualize the age-associated expression changes in pathways found significant by GSEA. Gene expression changes of aged vs. young stroma is represented in either red (up in aged) or green (down in aged). More intense color is used to show genes significantly changed with p-value <0.05. Grey color indicates no change while white indicates gene not present.

### White Blood cell collection and RNA extraction

Whole white blood cells samples were collected from young (4 month-old; n = 5) and old (24 month-old; n = 4) C57BL/6 mice by cardiac puncture under halothane anesthesia before sacrifice by cervical dislocation. Blood samples were kept on ice until fractionation. Blood was fractionated by centrifugation at 1400 rpm for 10 minutes at room temperature. Serum was removed, and the exposed white blood cells layer (or buffy coat) was carefully aspirated. The white blood cell preparations for each age group were pooled and treated with 10 ml of Hemolytic Buffer (NH_4_Cl, 8.3 mg/ml; NaHCO_3_ 1 mg/ml; Na_2_EDTA 0.4 mg/ml) preheated at 37°C and incubated at room temperature for 10 minutes. Cells were pellet at 750 rpm and washed twice with PBS. Samples were store frozen at −80°C until RNA isolation. Total RNA extraction was performed using Qiagen RNeasy kit, following the manufacturer's protocol. Total RNA was amplified one round and the amino-allyl UTP incorporated using the Ambion MessageAmp aRNA Kit (Ambion Inc, Austin, TX), according to the manufacturer's specifications. The amplified RNA was used as template for cDNA probe synthesis followed by hybridization to a 44K oligonucleotide microarray (Agilent, Inc). The Mouse Gold Standard was used as a reference RNA in order to compare the white blood cell and the aging transcriptional profiles.

### Quantitative RT-PCR

RNA from microdissected mouse prostate stroma and epithelium, and human prostate fibroblast (PSC27) were used as template for qRT-PCR. 200 ng of amplified RNAs or 20 µg of total RNAs, respectively, were used to generate cDNAs. SYBR GREEN real-time PCR was performed as previously described [71]. Primers to ribosomal protein S16 (for mouse samples) and RPL13 (for human samples) were used to normalize cDNA loading. The sequences of the primers used in this study are in Table S1 (supporting information).

### Immunohistochemistry

Formalin-fixed, paraffin-embedded mouse prostate tissue sections were deparaffinized, and endogenous peroxidase activity was blocked with 3% H_2_O_2_ for 8 min. Antigen was retrieved by steam heating with 10 mM citrate buffer (pH 6.0) for 20 min. Primary antibodies and working dilutions were as follows: Rat anti-CD3 (1∶500, Serotech MCA1477); Rat anti-B220 (1∶1000,Chemicon CBL1342); Rat anti-F4/80 (1:50, Serotech MCA497GA). After incubation of the primary antibody for 1 hour, slides were washed and incubated for 30 minutes with biotinylated species-specific secondary antibody (Biotin-SP conjugated Goat Anti-Rat IgG (H+L); 1∶200, Jackson ImmunoResearch 112-065-167), washed, and then incubated with avidin-peroxidase complex (ABC, Vector Laboratories) for 30 minutes and visualized using DAKO Dab system. The sections were dehydrated, and permanently mounted. Species-specific IgG isotype were added in lieu of primary antibody as controls, and these sections demonstrated no detectable staining. Immunoreactive cells with CD3, F4/80 and B220 antibodies were counted under 10X fields. The number of each cell is recorded separately with regards to its location (intraglandular vs. periglandular vs. interglandular) within each lobe.

### Immunofluorescence and Confocal Microscopy

OCT-embedded frozen prostates from five young (4 month-old) and five aged (24 month-old) mice were used for staining. Briefly, 7 µm and 30 µm sections, for light and confocal microscopy respectively, were fixed with 4% formaldehyde for 8 or 20 minutes, respectively. After fixation, the tissue sections were permeabilized with 0.1% Triton X-100 in Dulbecco's phosphate-buffered saline (PBS) for 10 minutes, followed by 3 washes with PBS. Tissue sections were exposed to a blocking solution consisting of 1X SuperBlock Reagent and 5% Normal Goat Serum in PBS for 1 hour. Next, the sections were treated with primary antibody in blocking solution overnight at 4°C. Primary antibodies and working dilutions were as follows: Type I collagen (1∶100, Rockland Inc. 600-401-103), vimentin (1∶700, Abcam. ab45939). After three subsequent washing steps (5 minutes each), the primary antibody was visualized with goat anti-rabbit IgG secondary antibody bound to Alexa Fluor 488 (Invitrogen, Carlsbad, CA) in blocking solution and incubated for 1 hour. For vimentin-smooth muscle staining, sections were simultaneously stained for smooth-muscle actin using anti-Smooth Muscle-Cy3 (1∶200, Sigma C6198) along with the secondary antibody. After four additional washing steps (5 minutes each), cover slips were mounted onto the microscope slides with anti-fade mountshield containing DAPI. Sections of 7 µM were examined by light-fluorescence microscopy. Sections of 30 µM were stained with Collagen Type I and visualized by confocal microscopy. In brief, stacks of confocal images were acquired on a Zeiss LSM 510 NLO confocal and two-photon microscope (Carl Zeiss, Inc. Thornwood, NY) fitted with a Zeiss 40x/1.3 PlanNeofluar oil immersion objective. The following laser lines and emission filters were used: DAPI: two photon excitation at 780 nm, 435–485 nm bandpass detection; 594 AlexaFluor: 543 nm excitation, 565–615 nm bandpass emission. Maximum intensity projections of selected confocal sections were obtained with Zeiss LSM software or ImageJ.

Collagen fiber appearance of prostatic stroma of anterior lobe in five young and old mice was compared using 4 images from each sample taken at 400X magnification. Qualitative criteria assessing the structure of collagen were devised as follows. The collagen fibers were classified as organized when the fibers had continuous, evenly spaced, mostly parallel pattern and disorganized when the architecture appeared haphazard. The individual fibers were defined as compact when the fibers were delicate, their thickness was even and swollen when the relative thickness increased and fibers became coarse. In addition, each fiber was identified as sharp when the edges of the fibers were smooth or fuzzy when irregularities were observed.

Images were independently scored by three individuals (including one pathologist) and a consensus was obtained.

### Electron Microscopy

For transmission EM, fragments of prostate lobes were fixed by immersion with 1/2 strength Karnovsky's fixative (2% Paraformaldehyde/2.5% Glutaraldehyde in 0.2 M Cacody-late buffer). After washing with 0.1 M Cacodylate buffer, fixed tissues were treated with 2% osmium tetroxide buffered in 0.2 M cacodylate buffer for 3 hours. Specimens were washed again and dehydrated in graded ethanol series (35% to 100% ethanol) and 2x propylene oxide and embedded in Epon resin. Ultrathin sections were cut and contrasted with uranyl acetate for 2 hrs, followed by lead citrate for 5 min. The samples were observed and evaluated with a JEOL 1230 transmission electron microscope. For scanning EM, whole prostate lobes (n = 5 young and n = 5 old) were fixed by immersion with 1/2 strength Karnovsky's fixative (2% Paraformaldehyde/2.5% Glutaraldehyde in 0.2 M Cacodylate Buffer). Fixed tissues were immersed in 10% sodium hydroxide for 6 days to remove cellular constituents (the solution was changed daily). The acellular preparations were then washed for several hours in distilled water. After rinsing, the samples were fixed in 1% tannic acid overnight, rinsed again in distilled water and post-fixed in 1% Osmium tetroxide for 3 hours. Specimens were then dehydrated in graded concentrations of ethanol, critical-point-dried, mount onto stubs and sputter coated with Au/Pd. The samples were evaluated and imaged with a JEOL JSM-5800 scanning electron microscope with an acceleration voltage of 15 kV, WD of 24.0 mm and tilt separation of 7–8°.

## Supporting Information

Figure S1 Cellular composition of prostatic glandular-adjacent stroma. Double immunofluorescent stain for smooth-muscle-actin (Red; C, F, I and L) and vimentin (green, B, E, H and K) demonstrating the prevalence of smooth-muscle cells (in red) in the glandular-adjacent stroma in both young (A,C,G, I) and old (J, L, D, F) prostates. Scatter fibroblast (in green) are also present in the glandular-adjacent stroma. A, D, G and J are merged images (Blue: DAPI, Red: smooth-muscle-actin, Green: vimentin).
(0.66 MB PDF)

Figure S2 Age-associated transcripts in prostate stroma. Heat map of differentially expressed genes (p<0.05) from microdissected glandular-adjacent stroma, using an independent set of 4 month-old (n = 12) and 24 month-old (n = 12) C57BL/6 mice and a microarray platform comprised of oligonucleotides complementary to ∼40,000 genes (Agilent). Gene symbols shown are the top 20 most up- and down-regulated genes. Heat map colors reflect fold ratio values between sample and reference pool and mean-centered across samples. Columns represent biological replicates from dorsal and anterior microdissected stroma for each age group. Rows represent individual genes. Values shown in red are relatively higher than the overall mean; values shown in green are relatively lower than the overall mean. Unpaired, two-sample t-tests were used to identify significant genes (p<0.05).
(0.09 MB PDF)

Figure S3 Age associated alterations in the stroma of immunodeficient mice housed in a barrier facility. Young (4 month-old; n = 3) and old (13 month-old; n = 3) ICR-SCID mice were housed in a barrier facility and kept in individual cages until sacrifice. A,B) Hematoxylin and eosin-stained sections of formalin-fixed prostate tissues. Note the smooth-muscle cells (arrows) appear less elongated and more rounded in the aged prostate with little evidence of cell orientation. C) Confirmation of Apod overexpression in prostates from aged ICR-SCID mice by qRT-PCR. RNA was extracted from the dorsal prostate lobes from ICR-SCID young (n = 3) and old (n = 3) mice and reverse transcribed using qRT-PCR with primers specific for Apod. S16 transcript expression levels were used to normalize the qRT-PCR data.
(0.25 MB PDF)

Figure S4 Expression of senescence-associated genes in prostate stroma. A) qRT-PCR for selected senescence-associated factors. RNA is from microdissected stroma from young and old mice. Pre-senescent (B) and senescent (C) prostatic smooth-muscle cells, demonstrating positive SA-β-Gal stain after H2O2 treatment. qRT-PCR for selected senescence associated secretory factors. RNA is from pre-senescent (pre-SEN) and senescent (SEN) mouse prostate smooth-muscle cells.
(0.02 MB PDF)

Figure S5 Expression of aging-associated genes in senescent cells. Heat map of age related changes in the prostate stroma compared to human in vitro senescence. The heat map represent the significantly differentially expressed genes from in vivo aged stroma (less than 25% FDR) that overlap with significantly altered genes (FDR <25%) in at least one human senescent data set. Red indicates increased expression; green indicates decreased expression; black represents no change in expression and grey represents no information on that gene.
(0.03 MB PDF)

Figure S6 Cytokine and Inflammatory Response Pathway. GenMAPP 2.1 (www.genmapp.org) was used to visualize the age associated changes in the Cytokine and Inflammatory Response Pathway. Gene expression changes of aged vs. young stroma is represented in either red (up in aged) or green (down in aged). More intense color is used to show genes significantly changed with p-value <0.05. Grey color indicates no change while white indicates gene not present.
(0.46 MB PDF)

Figure S7 Analysis of inflammatory cell types and numbers in young and aged prostate glands. Shown are the number of inflammatory cells present in young and old prostate glands according to each prostate lobe and anatomical location within lobe. The number of cells positive for each immune-cell marker (anti-F4/80, anti-CD3 and anti-B220, which recognize macrophages, T cell and B cells, respectively) were determined by quantitating the number of cells per 10X field in stained sections from young (4 months; n = 10) and old (24 months; n = 13) prostates. Inflammatory infiltrates were divided into three different categories: intraglandular infiltrates (inflammatory cells in contact with the glandular luminal epithelium); periglandular stromal infiltrates (inflammatory cells in contact with the smooth-muscle/fibroblastic cellular stroma); and interglandular infiltrates (inflammatory cells in the interglandular space).
(0.45 MB PDF)

Table S1 Primers sequence used for qRT-PCR
(0.07 MB PDF)
