# Supplementary figures and images for: The Effects of Aging on the Molecular and Cellular Composition of the Prostate Microenvironment
Source: PLoS One. 2010 Sep 1;5(9):e12501. doi: 10.1371/journal.pone.0012501 (PMC2931699; doi:10.1371/journal.pone.0012501)

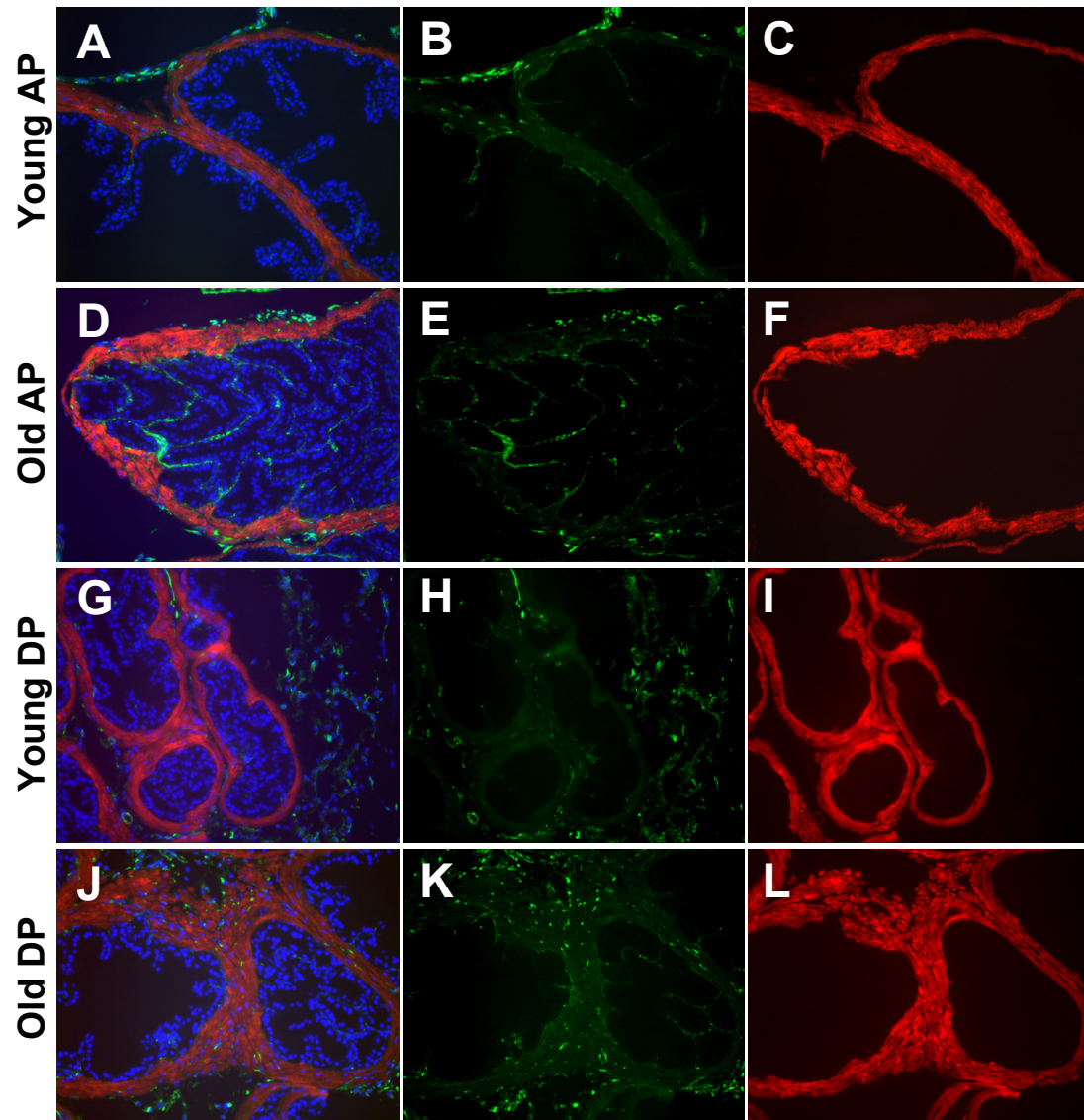

Figure S1. Bianchi-Frias et al., 2010

Supplement: Figure S1 — Cellular composition of prostatic glandular-adjacent stroma. Double immunofluorescent stain for smooth-muscle-actin (Red; C, F, I and L) and vimentin (green, B, E, H and K) demonstrating the prevalence of smooth-muscle cells (in red) in the glandular-adjacent stroma in both young (A,C,G, I) and old (J, L, D, F) prostates. Scatter fibroblast (in green) are also present in the glandular-adjacent stroma. A, D, G and J are merged images (Blue: DAPI, Red: smooth-muscle-actin, Green: vimentin). (0.66 MB PDF) [file pone.0012501.s001.pdf]

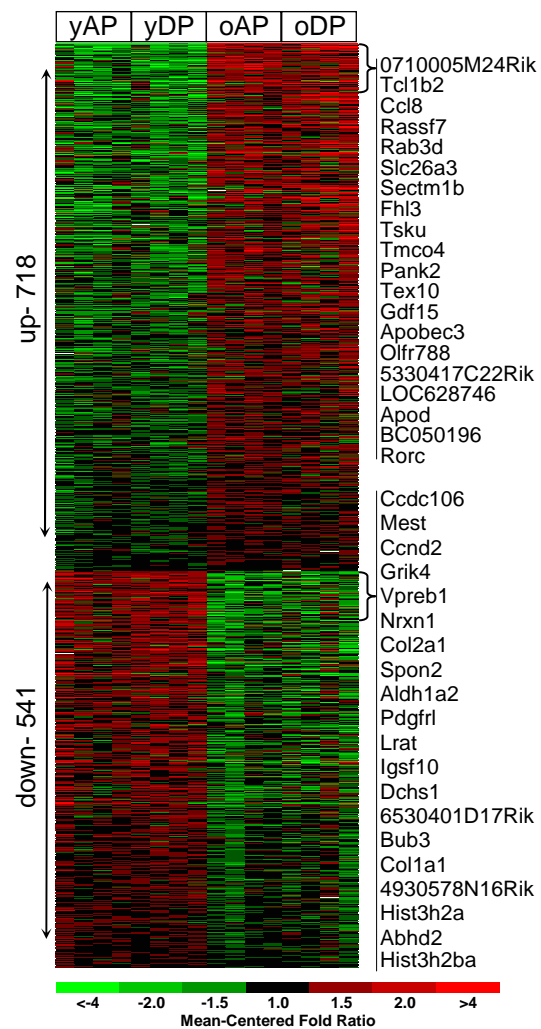

**Figure S2. Bianchi-Frias et al., 2010**

Supplement: Figure S2 — Age-associated transcripts in prostate stroma. Heat map of differentially expressed genes (p<0.05) from microdissected glandular-adjacent stroma, using an independent set of 4 month-old (n = 12) and 24 month-old (n = 12) C57BL/6 mice and a microarray platform comprised of oligonucleotides complementary to ∼40,000 genes (Agilent). Gene symbols shown are the top 20 most up- and down-regulated genes. Heat map colors reflect fold ratio values between sample and reference pool and mean-centered across samples. Columns represent biological replicates from dorsal and anterior microdissected stroma for each age group. Rows represent individual genes. Values shown in red are relatively higher than the overall mean; values shown in green are relatively lower than the overall mean. Unpaired, two-sample t-tests were used to identify significant genes (p<0.05). (0.09 MB PDF) [file pone.0012501.s002.pdf]

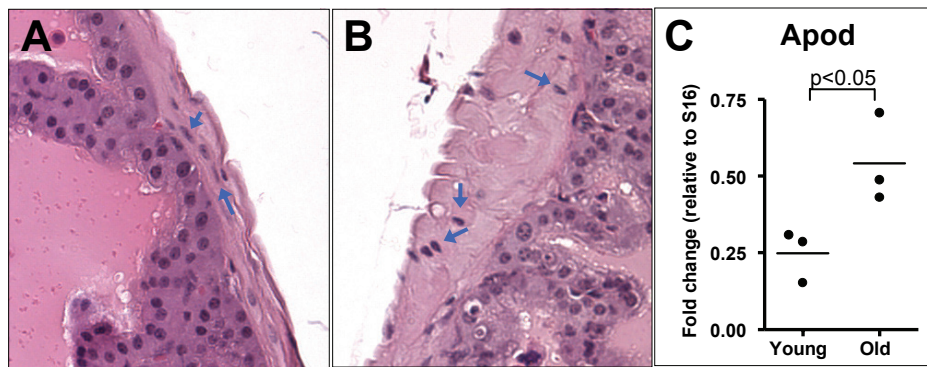

Figure S3. Bianchi-Frias et al., 2010

Supplement: Figure S3 — Age associated alterations in the stroma of immunodeficient mice housed in a barrier facility. Young (4 month-old; n = 3) and old (13 month-old; n = 3) ICR-SCID mice were housed in a barrier facility and kept in individual cages until sacrifice. A,B) Hematoxylin and eosin-stained sections of formalin-fixed prostate tissues. Note the smooth-muscle cells (arrows) appear less elongated and more rounded in the aged prostate with little evidence of cell orientation. C) Confirmation of Apod overexpression in prostates from aged ICR-SCID mice by qRT-PCR. RNA was extracted from the dorsal prostate lobes from ICR-SCID young (n = 3) and old (n = 3) mice and reverse transcribed using qRT-PCR with primers specific for Apod. S16 transcript expression levels were used to normalize the qRT-PCR data. (0.25 MB PDF) [file pone.0012501.s003.pdf]

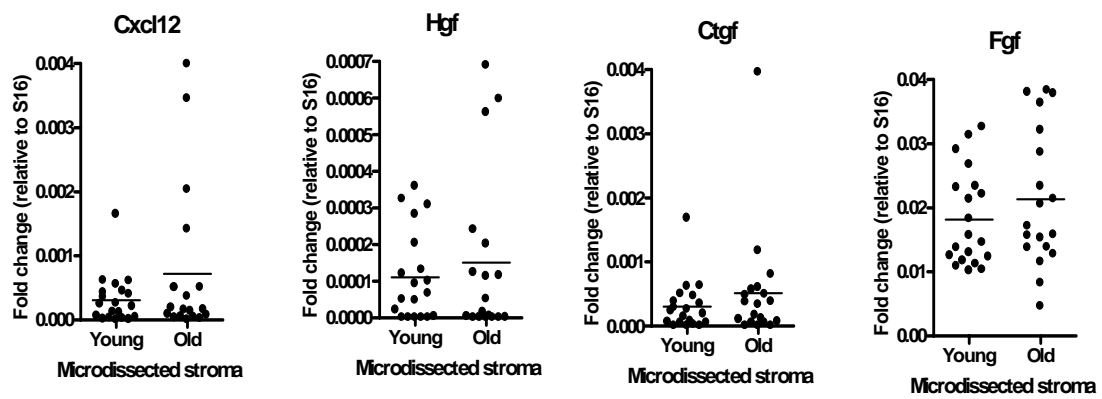

Figure S4. Bianchi-Frias et al., 2010

Supplement: Figure S4 — Expression of senescence-associated genes in prostate stroma. A) qRT-PCR for selected senescence-associated factors. RNA is from microdissected stroma from young and old mice. Pre-senescent (B) and senescent (C) prostatic smooth-muscle cells, demonstrating positive SA-β-Gal stain after H2O2 treatment. qRT-PCR for selected senescence associated secretory factors. RNA is from pre-senescent (pre-SEN) and senescent (SEN) mouse prostate smooth-muscle cells. (0.02 MB PDF) [file pone.0012501.s004.pdf]

## Cytokines and Inflammatory Response (BioCarta)

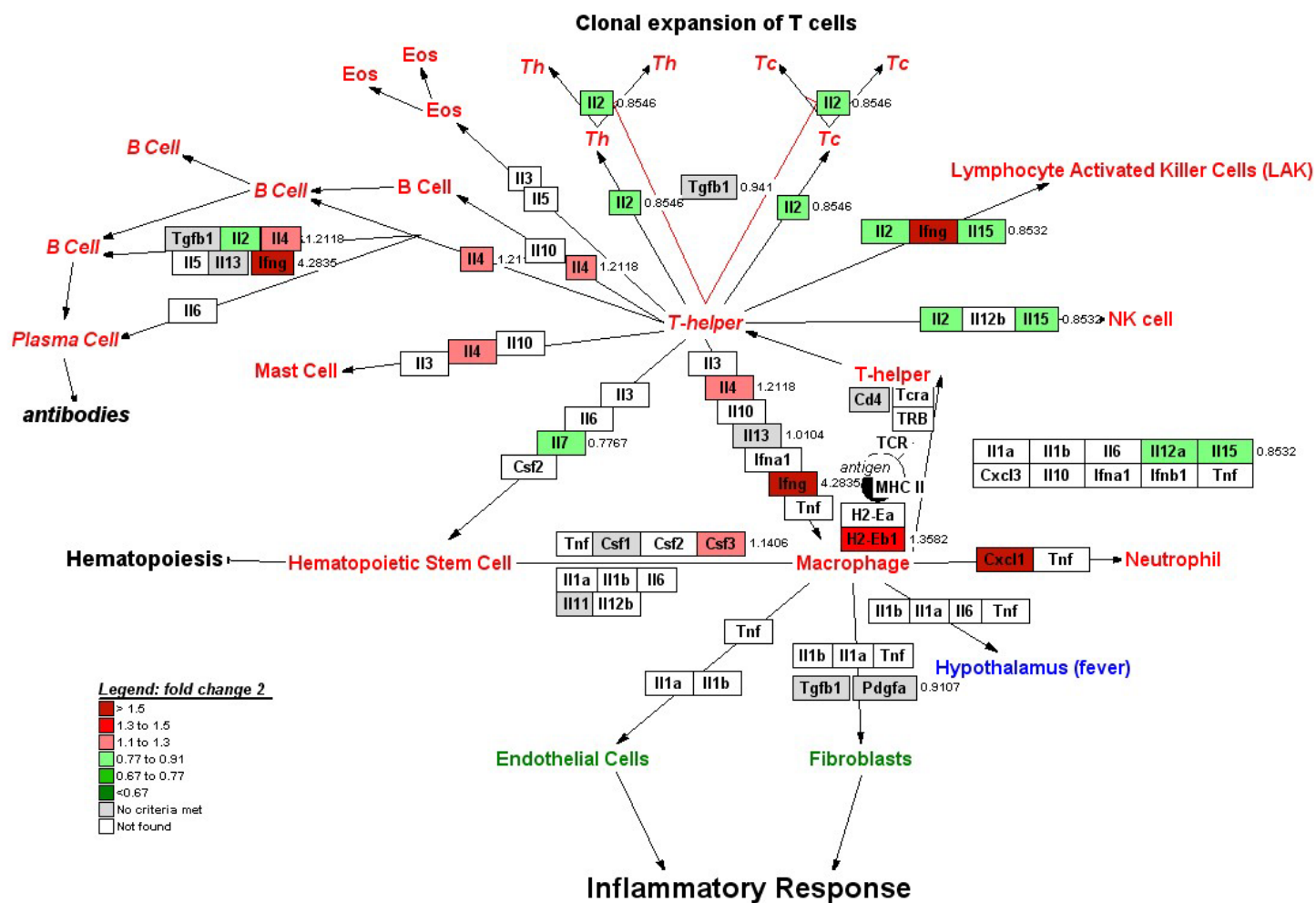

Figure S6. Bianchi-Frias et al., 2010

Supplement: Figure S6 — Cytokine and Inflammatory Response Pathway. GenMAPP 2.1 (www.genmapp.org) was used to visualize the age associated changes in the Cytokine and Inflammatory Response Pathway. Gene expression changes of aged vs. young stroma is represented in either red (up in aged) or green (down in aged). More intense color is used to show genes significantly changed with p-value <0.05. Grey color indicates no change while white indicates gene not present. (0.46 MB PDF) [file pone.0012501.s006.pdf]
