# Supplementary material for: The Effects of Aging on the Molecular and Cellular Composition of the Prostate Microenvironment
Source: PLoS One. 2010 Sep 1;5(9):e12501. doi: 10.1371/journal.pone.0012501 (PMC2931699; doi:10.1371/journal.pone.0012501)
Supplement: Figure S5 — Expression of aging-associated genes in senescent cells. Heat map of age related changes in the prostate stroma compared to human in vitro senescence. The heat map represent the significantly differentially expressed genes from in vivo aged stroma (less than 25% FDR) that overlap with significantly altered genes (FDR <25%) in at least one human senescent data set. Red indicates increased expression; green indicates decreased expression; black represents no change in expression and grey represents no information on that gene. (0.03 MB PDF) [file pone.0012501.s005.pdf]

HUGO	Description	FDR (%)	ASH 18K	ASH	ASB	RS	P16	RAS
APOD	apolipoprotein D	0.0	*	na	na	na	na	na
RAB3D	RAB3D, member RAS oncogene family	3.8					*	
IL7R	interleukin 7 receptor	5.9		*				
EFHD1	EF-hand domain family, member D1	5.9					*	
INADL	InaD-like (Drosophila)	7.1				*	*	
PERP	PERP, TP53 apoptosis effector	8.1	na	*		*		*
CCL7	chemokine (C-C motif) ligand 7	8.1					*	*
WDR45L	WDR45-like	9.6					*	
GDF15	growth differentiation factor 15	12.5	*	na		na	na	na
ATF3	activating transcription factor 3	15.6	*	na	na	na	na	na
SV2B	synaptic vesicle glycoprotein 2B	15.6	na					
PANK2	pantothenate kinase 2 (Hallervorden-Spatz syndrome)	17.2				*	*	*
TMPRSS2	transmembrane protease, serine 2	17.2					*	
TLR1	toll-like receptor 1	17.2					*	na
STAT1	signal transducer and activator of transcription 1, 91kDa	18.4		*	*	*	*	*
B2M	beta-2-microglobulin	18.4		*	*		*	*
LRPAP1	low density lipoprotein receptor-associated protein 1	18.4			*	*	*	
DDB2	damage-specific DNA binding protein 2, 48kDa	18.4			*	*		
SCYL3	SCY1-like 3 (S. cerevisiae)	18.4				*	*	*
LGALS3	lectin, galactoside-binding, soluble, 3 (galectin 3)	18.4		*			*	
BDNF	brain-derived neurotrophic factor	18.4				*	*	*
IER3	immediate early response 3	18.4						*
PARP8	poly (ADP-ribose) polymerase family, member 8	18.4					*	na
SLC16A1	solute carrier family 16, member 1	18.4					*	*
THBS1	thrombospondin 1	18.4		na	na	*	na	na
HBP1	HMG-box transcription factor 1	21.8		*			*	
PRDX6	peroxiredoxin 6	21.8				*	*	
EHF	ets homologous factor	21.8	na			*	*	*
CXCL16	chemokine (C-X-C motif) ligand 16	21.8				*	*	
TAP1	transporter 1, ATP-binding cassette, sub-family B	21.8			*		na	na
SOX9	SRY (sex determining region Y)-box 9	23.8					*	*
SLC25A25	solute carrier family 25 , member 25	23.8					*	
USP33	ubiquitin specific peptidase 33	23.8					*	
DNAJC10	DnaJ (Hsp40) homolog, subfamily C, member 10	23.8				*	na	na
NR4A1	nuclear receptor subfamily 4, group A, member 1	23.8					*	
CCND2	cyclin D2	9.6			*	*	*	*
MEST	mesoderm specific transcript homolog (mouse)	9.6					na	na

Column groups: Mouse LCM stroma (Young, Old); Human Senescent Prostate stroma cells (ASH 18K, ASH, ASB, RS, P16, RAS); Human <25% FDR (ASH 18K, ASH, ASB, RS, P16, RAS).

Figure S5. Bianchi-Frias et al., 2010
