# Supplementary material for: The Effects of Aging on the Molecular and Cellular Composition of the Prostate Microenvironment
Source: PLoS One. 2010 Sep 1;5(9):e12501. doi: 10.1371/journal.pone.0012501 (PMC2931699; doi:10.1371/journal.pone.0012501)
Supplement: Figure S7 — Analysis of inflammatory cell types and numbers in young and aged prostate glands. Shown are the number of inflammatory cells present in young and old prostate glands according to each prostate lobe and anatomical location within lobe. The number of cells positive for each immune-cell marker (anti-F4/80, anti-CD3 and anti-B220, which recognize macrophages, T cell and B cells, respectively) were determined by quantitating the number of cells per 10X field in stained sections from young (4 months; n = 10) and old (24 months; n = 13) prostates. Inflammatory infiltrates were divided into three different categories: intraglandular infiltrates (inflammatory cells in contact with the glandular luminal epithelium); periglandular stromal infiltrates (inflammatory cells in contact with the smooth-muscle/fibroblastic cellular stroma); and interglandular infiltrates (inflammatory cells in the interglandular space). (0.45 MB PDF) [file pone.0012501.s007.pdf]

Macrophages (F4/80 marker)

Intraglandular (associated w/ epithelial cells)								Periglandular (associated w/ fibroblasts/SM-cells)								Interglandular (NOT associated w/ epithelial or fibroblasts/SM-cells)							
Ventral lobe		*Lateral lobe*		*Dorsal lobe*		*Anterior lobe*		*Ventral lobe*		*Lateral lobe*		*Dorsal lobe*		*Anterior lobe*		*Ventral lobe*		*Lateral lobe*		*Dorsal lobe*		*Anterior lobe*	
Young	Old	Young	Old	Young	Old	Young	Old	Young	Old	Young	Old	Young	Old	Young	Old	Young	Old	Young	Old	Young	Old	Young	Old
1	0	1	0	0	3	1	na	7	0	6	3	3	8	7	na	36	35	70	27	59	60	31	na
0	5	0	2	1	9	1	8	4	26	4	6	5	18	5	68	75	58	40	45	60	86	29	85
5	na	3	10	0	11	20	11	19	na	9	6	5	4	8	9	58	na	41	40	16	40	24	64
	2		na	10	na	8	35	3	17	4	na	3	na	3	24	50	27	38	na	33	na	16	49
4	7	5	75	12	18	13	47	0	2	0	6	2	23	1	14	32	77	28	120	12	40	9	43
6	19	5	8	7	10	7	28	4	11	2	3	4	4	6	52	50	34	31	18	24	15	19	210
2	15	2	2	6	na	4	70	2	6	1	9	5	na	2	24	20	37	17	48	10	na	10	120
0	15	0	12	10	5	5	34	6	1	3	8	7	0	5	27	43	45	9	61	11	34	30	42
2	na	0	3	1	10	0	na	2	na	2	10	3	2	2	na	89	na	52	77	83	50	30	na
	na		na		7		6		na		na		1		3		na		na		20		50
	7		17		3		18		2		6		4		7		44		58		22		39
	12		2		1		17		3		3		14		21		88		59		92		127
	6		0		9		20		2		4		9		15		22		39		36		14

T cells (CD3 marker)

Intraglandular (associated w/ epithelial cells)								Periglandular (associated w/ fibroblasts/SM-cells)								Interglandular (NOT associated w/ epithelial or fibroblasts/SM-cells)							
Ventral lobe		*Lateral lobe*		*Dorsal lobe*		*Anterior lobe*		*Ventral lobe*		*Lateral lobe*		*Dorsal lobe*		*Anterior lobe*		*Ventral lobe*		*Lateral lobe*		*Dorsal lobe*		*Anterior lobe*	
Young	Old	Young	Old	Young	Old	Young	Old	Young	Old	Young	Old	Young	Old	Young	Old	Young	Old	Young	Old	Young	Old	Young	Old
0	0	0	0	0	0	0	na	0	0	0	0	0	2	0	na	0	5	0	7	3	7	1	na
0	1	0	0	1	4	1	36	0	1	0	0	5	0	0	3	1	3	0	11	0	1		2
0	0	2	3	0	9	0	11	1	na	0	0	0	0	0	0	2	0	0	0	0	4	1	28
0	24	4	na	2	na	1	16	1	3	0	na	1	na	0	22	1	5	1	na	1	na	4	30
4	15	3	55	1	125	4	200	1	0	0	0	0	0	0	1	1	3	1	22	0	4	2	2
0	8	4	10	3	18	0	17	0	9	0	5	0	1	0	28	0	163	0	124	0	8	1	630
1	10	1	12	0	na	1	62	1	4	2	3	0	na	0	7	0	92	5	49	3	na	0	800
3	9	0	22	10	16	3	90	0	0	0	5	0	3	1	2	0	8	0	28	0	20	0	23
2	na	4	0	2	na	4	na	1	na	5	0	0	0	2	na	1	na	2	1	0	9	3	na
0	na	1	na	0	42	7	18	0	na	0	na	0	1	0	2	0	na	0	na	0	0	3	13
2	3	1	4	2	6	0	28	0	0	0	1	0	0	1	2	0	0	0	4	2	2	1	33
	8		0		3		11		0		0		1		3		3		1		14		30
	5		1		0		16		0		2		0		2		2		13		26		9

B cells (B220 marker)

Intraglandular (associated w/ epithelial cells)								Periglandular (associated w/ fibroblasts/SM-cells)								Interglandular (NOT associated w/ epithelial or fibroblasts/SM-cells)							
Ventral lobe		*Lateral lobe*		*Dorsal lobe*		*Anterior lobe*		*Ventral lobe*		*Lateral lobe*		*Dorsal lobe*		*Anterior lobe*		*Ventral lobe*		*Lateral lobe*		*Dorsal lobe*		*Anterior lobe*	
Young	Old	Young	Old	Young	Old	Young	Old	Young	Old	Young	Old	Young	Old	Young	Old	Young	Old	Young	Old	Young	Old	Young	Old
0	0	0	0	0	0	0	na	na	0		0	0	0	0	na	na	0	0	5		0	0	na
	0		0		0		16		0	0	2		0		12		0		38	0	0		8
0	0	0	0	0	0	0	0	0	0		0	0	0	0	0	0	0	0	0		0	0	0
0	0	0	na	0	na	0	5	0	0	0	na	0	na	0	0	0	9	0	na	0	na	0	200
0	0	0	0	0	0	0	0	0	0	0	0	0	0	0	0	0	3	0	0	0	0	0	0
?	0	?	0	0	0	0	3	0	0	0	0	0	0	0	2	0	0	0	58	0	0	0	18
0	0	0	0	0	na	0	0	0	0	0	0	0	na	0	3	0	28	0	50	0	na	0	600
0	0	0	0	0	0	0	2	0	0	0	0	0	0	0	2	0	5 iv	0	1	0	0	0	5
0	na	0	0	0	0	0	5	0	na	0	0	0	0	1	na	0	na	15	4	0	19	0	na
0	na	0	na	0	0	0	2	0	na	0	na	0	0	0	0	0	na	0	na	0	0	0	0
0	0	0	0	0	0	1	0	0	0	0	0	0	0	1	2	0	1	0	0	0	13	0	57
	0		0		0		0		0	0	0		0		1		2		2	0	10		80
	0		0		0		6		0		0		0		5		0		41		40		12

Figure S7. Bianchi-Frias et al., 2010
