# Supplementary material for: The Effects of Aging on the Molecular and Cellular Composition of the Prostate Microenvironment
Source: PLoS One. 2010 Sep 1;5(9):e12501. doi: 10.1371/journal.pone.0012501 (PMC2931699; doi:10.1371/journal.pone.0012501)
Supplement: Table S1 — Primers sequence used for qRT-PCR (0.07 MB PDF) [file pone.0012501.s008.pdf]

Table S1. Primers sequence

Gene Symbol	Primer sequence
mS16 forward	5'- AGGAGCGATTTGCTGGTGTGGA -3'
mS16 reverse	5'- GCTACCAGGCCTTTGAGATGGA -3'
mCcl8 forward	5'- AGAGACAGCCAAAGCTGGAA -3'
mCcl8 reverse	5'- CAGGCACCATCTGCTTGTAA -3'
mApoD forward	5'- CCAAACAGAGCAACGTCTCAGA -3'
mApoD reverse	5'- GAAGAAGGTGGTGCAGGAGTACA -3'
hAPOD forward	5'- GCCTGCCAAGCTGGAAGTT -3'
hAPOD reverse	5'- GGCCAGGATCCAGTACGGT -3'
hRPL13 forward	5'- CCTGGAGGAGAAGAGGAAAGAGA -3'
hRPL13 reverse	5'- TTGAGGACCTCTGTGTATTTGTCAA -3'
mCol1a2 forward	5'- ACCCCAGCGAAGAACTCATACA -3'
mCol1a2 reverse	5'- CCATTGATAGTCTCTCCTAACCAGACA -3'
Col1a1 forward	5'- GCCTCAGCCACCTCAAGAGA -3'
Col1a1 reverse	5'- GGCTGCGGATGTTCTCAATC -3'
Col3a1 forward	5'- TTGATGTGCAGCTGGCATTC -3'
Col3a1 reverse	5'- GCCACTGGCCTGATCCATAT -3'
Col4a1 forward	5'- GCCAAGTGTGCATGAGAAGA -3'
Col4a1 reverse	5'- GGAGGGAGAAGAGGACAAGG -3'
